# Broadly neutralizing antibodies derived from the earliest COVID-19 convalescents protect mice from SARS-CoV-2 variants challenge

**DOI:** 10.1038/s41392-023-01615-0

**Published:** 2023-09-14

**Authors:** Qianyun Liu, Haiyan Zhao, Zhiqiang Li, Zhen Zhang, Rui Huang, Mengxue Gu, Ke Zhuang, Qing Xiong, Xianying Chen, Weiyi Yu, Shengnan Qian, Yuzhen Zhang, Xue Tan, Muyi Zhang, Feiyang Yu, Ming Guo, Zhixiang Huang, Xin Wang, Wenjie Xiang, Bihao Wu, Fanghua Mei, Kun Cai, Limin Zhou, Li Zhou, Ying Wu, Huan Yan, Sheng Cao, Ke Lan, Yu Chen

**Affiliations:** 1 State Key Laboratory of Virology, Institute for Vaccine Research, College of Life Sciences, Wuhan University, Wuhan, 430072 China; 2 Department of Thoracic Surgery, Renmin Hospital, Wuhan University, Wuhan, China; 3 CAS Key Laboratory of Special Pathogens, Wuhan Institute of Virology, Center for Biosafety Mega-Science, Chinese Academy of Sciences, Wuhan, 430071 P. R. China; 4 Animal Biosafety Level-III Laboratory/Institute for Vaccine Research, Wuhan University, Wuhan, China; 5 State Key Laboratory of Virology and Hubei Province Key Laboratory of Allergy and Immunology, Institute of Medical Virology, TaiKang Medical School, Wuhan University, Wuhan, 430072 China; 6 Hubei Center for Disease Control and Prevention, Wuhan, 430079 China; 7 Maternal and Child Health Hospital of Hubei Province, Tongji Medical College, Huazhong University of Science and Technology, Wuhan, China

**Keywords:** Adaptive immunity, Microbiology

## Abstract

Coronavirus disease 2019 (COVID-19) was first reported three years ago, when a group of individuals were infected with the original SARS-CoV-2 strain, based on which vaccines were developed. Here, we develop six human monoclonal antibodies (mAbs) from two elite convalescents in Wuhan and show that these mAbs recognize diverse epitopes on the receptor binding domain (RBD) and can inhibit the infection of SARS-CoV-2 original strain and variants of concern (VOCs) to varying degrees, including Omicron strains XBB and XBB.1.5. Of these mAbs, the two most broadly and potently neutralizing mAbs (7B3 and 14B1) exhibit prophylactic activity against SARS-CoV-2 WT infection and therapeutic effects against SARS-CoV-2 Delta variant challenge in K18-hACE2 KI mice. Furthermore, post-exposure treatment with 7B3 protects mice from lethal Omicron variants infection. Cryo-EM analysis of the spike trimer complexed with 14B1 or 7B3 reveals that these two mAbs bind partially overlapped epitopes onto the RBD of the spike, and sterically disrupt the binding of human angiotensin-converting enzyme 2 (hACE2) to RBD. Our results suggest that mAbs with broadly neutralizing activity against different SARS-CoV-2 variants are present in COVID-19 convalescents infected by the ancestral SARS-CoV-2 strain, indicating that people can benefit from former infections or vaccines despite the extensive immune escape of SARS-CoV-2.

## Introduction

Severe acute respiratory syndrome coronavirus 2 (SARS-CoV-2) emerged around three years ago and generated a worldwide health and economic crisis.^1–3^ More than 700 million individuals have been infected by SARS-CoV-2, and more than 6.9 million deaths have been reported (https://covid19.who.int/). SARS-CoV-2 continues to mutate and leads to the emergence of several VOCs (Alpha, Beta, Gamma, Delta, and Omicron), which have posed significant challenges to current vaccines and therapeutic mAbs,^4,5^ as the earlier generation of vaccines on the market were designed from the original strain. Multiple studies showed that the efficacy of some vaccines against viral variants is lower than against the ancestral strain, and breakthrough infections have been observed in naturally infected individuals and vaccinees.^6–10^ Despite this compromising effect, when exposed to the new SARS-CoV-2 variants, most people with a history of SARS-CoV-2 infection or vaccination showed less severe disease and hospitalization rates compared to unvaccinated and SARS-CoV-2-naïve individuals.^11,12^

The Spike (S) glycoprotein locates on the viral surface and interacts with hACE2 for viral entry.^3^ The S protein consists of the S1 and S2 subunits, and the S1 subunit can be further divided into the N-terminal domain (NTD), the RBD, and the sub-domains 1 and 2. The RBD of SARS-CoV-2 S adopts two distinct conformations, termed “up” and “down”,^4^ and the “up” position is believed to be required for hACE2 binding. The S2 subunit comprises a conserved fusion peptide that is crucial for viral fusion with cellular membranes after RBD binding by hACE2.^13,14^ mAbs against SARS-CoV-2 have been discovered to target both S1 and S2 subunits.^5^ S2-binding antibodies generally neutralize SARS-CoV-2 with lower potency, although some mAbs showed relatively broadly neutralizing activities against alpha- and beta-coronaviruses.^5,15–18^ The most potent mAbs target on the RBD of S1 and RBD-targeted antibodies have been recently categorized into seven groups (RBD-1 to RBD-7) based on Hastie’s system.^19^

Antibody genes usage to distinct pathogens varies significantly, and several commonly used germline genes for human mAbs to SARS-CoV-2 have been discovered. Published studies showed that heavy chain germlines VH3-30, VH3-30-3, VH3-53, VH3-66, VH3-23, VH1-2, VH1-58, and VH1-69 are the most frequently used genes for mAbs targeting SARS-CoV-2 Spike.^20–22^ The same germline-derived mAbs may have distinct epitopes and binding models on the same antigen. For example, mAbs encoded by VH1-69 recognize diverse domains of the S protein, including RBD, NTD, and S2.^20^ Even on the RBD, VH1-69-derived neutralizing mAb LY-CoV555 targets the peak of the receptor binding motif (RBM), grouped into the RBD-2 community.^23^ However, mAb S2X259 encoded by VH1-69 recognizes the outer face of the RBD, classified into the RBD-4 community.^24^ The molecular basis of public antibody responses has been extensively studied, while this feature of other rarely used antibody genes against SARS-CoV-2 infection still needs to be investigated.

Almost all patients in Wuhan were infected with the original SARS-CoV-2 strain, without exposure to distinct emerged variants at that time.^7^ The study of the humoral immune response of these Wuhan convalescents is important for understanding antibody immunity and protection against SARS-CoV-2 infection. Previously, we evaluated the antibody response of Wuhan’s convalescents at the polyclonal level using serum and noticed that the serum of a few convalescents (group 1, relatively resistant) displayed broad neutralizing potency against SARS-CoV-2 variants.^6^ Here, we isolate a panel of mAbs from two convalescents of group 1, and investigate the antibody immune response of these earliest convalescences at the monoclonal antibody level.

## Results

### Neutralizing monoclonal antibodies from convalescents can prevent SARS-CoV-2 infection in vivo

To obtain neutralizing mAbs from convalescents, we collected peripheral blood mononuclear cells (PBMCs) from the two earliest Wuhan COVID-19 convalescents at one year after SARS-CoV-2 WT infection. PBMCs were stained with Biotin-labeled S1 protein to sort antigen-specific memory B cells (MBCs), and a total of 226 S1^+^ single MBCs were successfully sorted into 96-well plates with frequencies of 0.70%. After screening 113 successfully expressed antibodies by SARS-CoV-2 pseudovirus neutralization assay, six potent neutralizing antibodies (3C4, 4G4, 7B3, 12G4, 13B2, and 14B1) were selected for further investigation.

**Fig. 1:**
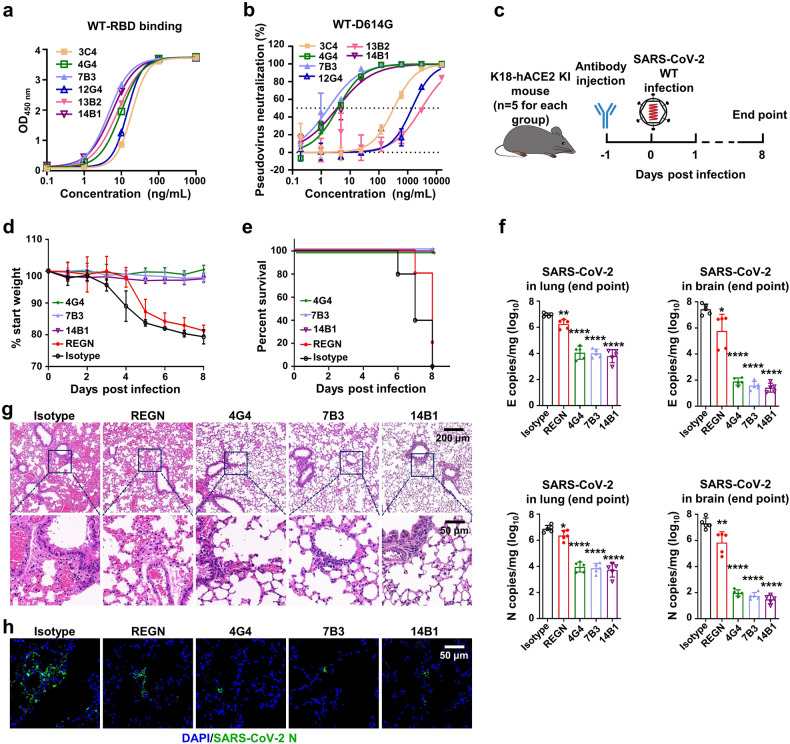
Monoclonal antibodies 7B3 and 14B1 can neutralize SARS-CoV-2 in vitro and in vivo. **a** The RBD binding assay of mAbs by ELISA. **b** The pseudovirus neutralization assay of mAbs against SARS-CoV-2 WT-D614G. **c** Time schedule for antibody protection in vivo. 12–18-week-old K18-hACE2 mice (n = 5 for each group) were injected 10 mg/kg antibodies at dpi −1, and were intranasally infected with 250 PFU/mL SARS-CoV-2 WT strain viruses each at dpi 0. Endpoint is at dpi 8 or when mice dead or reach 80% body weight, and the mice were euthanized and samples at endpoint. **d** Body weight changes. **e** Survival rate. **f** The SARS-CoV-2 RNA (E and N gene) copy number in lungs and brains. Unpaired *t* test. *p < 0.05; **p < 0.01; ****p < 0.0001. **g** The H&E stain of lungs. Scar bar, 200 μm in the up row, 50 μm in the down row. **h** The immunofluorescence analysis of SARS-CoV-2 N protein in the lungs. Nuclear DNA is stained by DAPI (4’,6-diamidino-2-phenylindole). Scar bar, 50 μm

The enzyme-linked immunosorbent assay (ELISA) results showed that all six mAbs were able to bind the RBD of SARS-CoV-2 Spike (Fig. 1a), even though S1 was used as the bite in MBCs sorting. We speculated that the antibodies that bound to RBD could more efficiently block the interaction between Spike and hACE2 and outcompeted in the initial screen of pseudovirus neutralization. Meanwhile, the 50% effective concentration (EC_50_) of these mAbs against SARS-CoV-2 pseudovirus (carrying the S of the SARS-CoV-2 WT strain with D614G mutation, WT-D614G) was determined, and three mAbs showed potent neutralizing activity. The EC_50_ values of 4G4, 7B3, and 14B1 were 3.7 ng/mL, 1.8 ng/mL, and 3.5 ng/mL, respectively (Fig. 1b).

Next, we evaluated the prophylactic effects of 4G4, 7B3, and 14B1 against SARS-CoV-2 infection in K18-hACE2 knock-in (KI) mice, which are highly susceptive to SARS-CoV-2. 12 ~ 18-week-old K18-hACE2 KI mice were intraperitoneally (i.p.) injected with 10 mg/kg mAbs on day −1 and then were infected intranasally with 250 PFU/mL SARS-CoV-2 WT viruses at day 0. REGN10933, which recognizes RBD and is an effective component of FDA-proved mAbs cocktail (REGN-COV2), was included as the positive control. The mice treated with the isotype control dramatically lost body weight from 3 dpi, and all succumbed to infection by 8 dpi. However, mice treated with 4G4, 7B3, or 14B1 all survived, and no significant weight change was observed (Fig. 1c–e). The viral loads of SARS-CoV-2 in the lungs and brains of K18-hACE2 KI mice were also determined. 4G4, 7B3, and 14B1 significantly decreased the viral loads in the lungs and brains compared to the isotype control (isotype) mAb (Fig. 1f). Lung pathology analysis showed that SARS-CoV-2 caused severe interstitial pneumonia characterized by a large number of inflammatory cells infiltrating the lungs and the wide thickening and rupture of the alveolar septum in the isotype group. In contrast, no obvious lesions were observed in mice injected with 4G4, 7B3, or 14B1 (Fig. 1g). Meanwhile, a large amount of SARS-CoV-2 antigen (Nucleocapsid, N protein, green) was detected in the lungs of mice treated with the isotype control, but little N protein was observed in mice treated with our mAbs (Fig. 1h). These results indicated that 4G4, 7B3, and 14B1 can efficiently protected the K18-hACE2 KI mice from SARS-CoV-2 WT infection.

### Epitope competition analyses

**Fig. 2:**
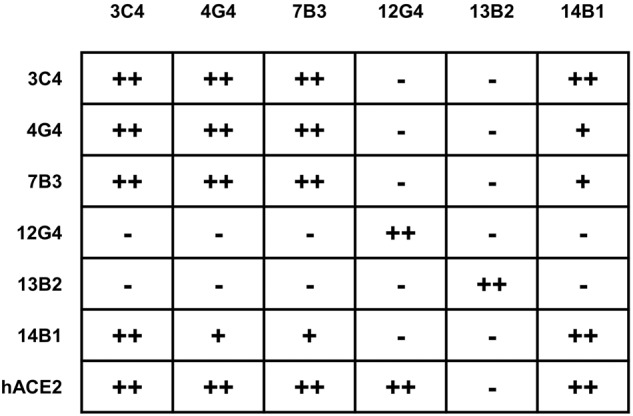
The epitope competition between mAbs. The mAbs in row were loaded onto protein A biosensors, and WT-RBD was then associated. The second mAbs or hACE2 were then loaded to detect the competition relationships (details in Supplementary Fig. 1). − no competition, + partial competition, ++ strong competition

We next quantitatively measured the binding capacity of mAbs for SARS-CoV-2 WT-RBD through biolayer interferometry (BLI). The kinetic binding affinity (*K*_*D*_) values of 7B3 and 14B1 were 2.6 × 10^–12^ mol/L and 3.1 × 10^–12^ mol/L, respectively, consistent with their high neutralizing activity against WT-D614G pseudovirus (Table 1). Receptor blockage is reported as the primary strategy for neutralizing antibodies to prevent SARS-CoV-2 infection. We further investigated the competition capacity of our mAbs and hACE2 for RBD binding by BLI. This analysis showed that the six mAbs recognized three major epitopes on RBD. Except for 13B2, all five mAbs blocked the binding of hACE2 to RBD (Supplementary Fig. 1a), partly explaining the inefficient neutralizing activity of 13B2 against SARS-CoV-2. 12G4 competes with hACE2, but the binding of 12G4 to RBD did not affect the binding of RBD by the other five mAbs. The highly potent neutralizing mAb 14B1 partially competed with 4G4 and 7B3, but severely blocked the interaction of RBD with 3C4. Taking the neutralizing activity into consideration, we propose that the combination of 7B3 and 14B1 has the potential to be used as antibody cocktails (Fig. 2 and Supplementary Fig. 1b).

**Table 1:** Characteristics of Anti-SARS-CoV-2 mAbs

mAb	source	Germline of Heavy Chain	Germline of light Chain	Affinity for WT-RBD *K*_*D*_ (nM)	EC_50_ of pseudovirus neutralization for SARS-CoV-2 WT-D614G (ng/mL)
3C4	PBMC of Convalescent 1	VH3–9*01	VK3–11*01	33.93	328.9
4G4		VH4–39*08	VL1–40*01	0.4685	3.748
7B3	PBMC of Convalescent 2	VH1–69*09	VK3–15*01	0.0026	1.759
12G4		VH1–18*01	VL6–57*02	2.364	1409
13B2		VH5–51*01	VL1–44*01	0.0014	3076
14B1		VH3–43*02	VK3–15*01	0.0031	3.511

### Cross-variant neutralization against major SARS-CoV-2 variants

**Fig. 3:**
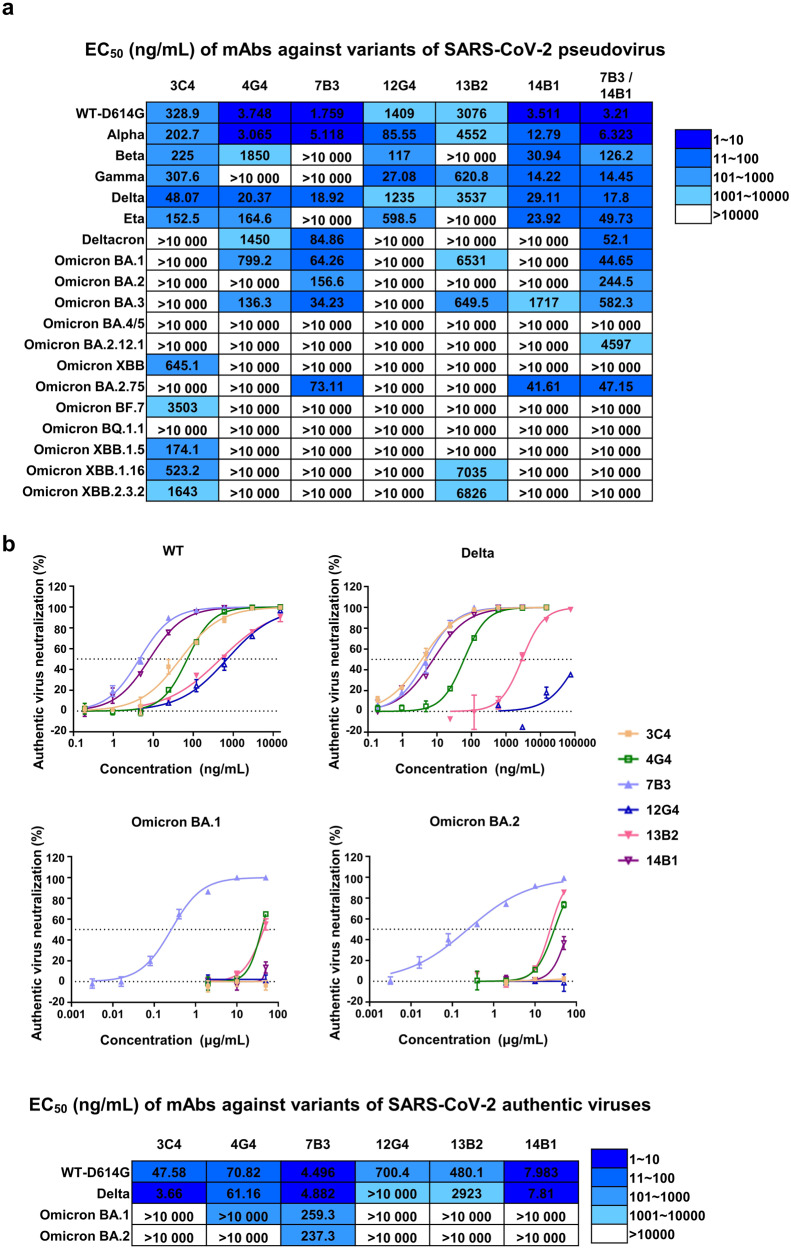
The neutralizing ability of mAbs against SARS-CoV-2 pseudovirus and authentic virus. **a** The half effective concentration (EC_50_) of mAbs against pseudovirus of different SARS-CoV-2 variants. **b** The authentic SARS-CoV-2 neutralization of mAbs through plaque reduction assay

SARS-CoV-2 has continued to mutate since its outbreak at the end of 2019, and there are five variants of concern so far, namely, Alpha (B.1.1.7), Beta (B.1.351), Gamma (P.1), Delta (B.1.617.2), and Omicron (B.1.1.529, BA.1, BA.2, BA.3, BA.4, BA.5, XBB, BA.2.75, BA.5.2, BF.7, XBB.1.5, XBB.1.16, and XBB.2.3.2). The amino acid alterations in the S protein of the variants are summarized in Supplementary Fig. 8. We further evaluate the binding capacity of mAbs against RBD of SARS-CoV-2 Delta, BA.1, and XBB.1.5 through BLI and ELISA, and found out that all six mAbs bind to WT- and- Delta-RBD with varying degrees (Supplementary Fig. 2). Then, the neutralizing activity of these six mAbs against several SARS-CoV-2 variants were investigated, including five VOCs, Deltacron, and Eta by pseudovirus neutralization assay and found that 7B3 potently neutralized the Alpha and Delta variants with EC_50_ values of 5.118 ng/mL and 18.92 ng/mL, respectively, but 7B3 completely lost its neutralizing activity against the Beta, Gamma, and Eta variants. Meanwhile, 14B1 showed potent and cross-variant neutralizing activity: its EC_50_ against Alpha, Beta, Gamma, Delta, and Eta variants were 12.79 ng/mL, 30.94 ng/mL, 14.22 ng/mL, 29.11 ng/mL and 23.92 ng/mL, respectively (Fig. 3a and Supplementary Fig. 3).

The Omicron variant, which is currently the dominant variant around the world, has many subvariants, all of which have extensive immune escape ability. We further tested our antibodies against multiple Omicron subvariants: BA.1, BA.2, BA.3, BA.4/5, BA.2.12.1, XBB, BA.2.75, BF.7, BQ.1.1, XBB.1.5, XBB.1.16, and XBB.2.3.2. Encouragingly, 7B3 neutralized BA.1, BA.2, BA.3, and BA.2.75 efficiently with EC_50_ values of 64.26 ng/mL, 156.6 ng/mL, 34.23 ng/mL and 73.11 ng/mL, respectively. With respect to BA.4/5, BA.2.12.1, BF.7, and BQ.1.1, all six mAbs lost their neutralizing activity (Fig. 3a and Supplementary Fig. 3). Interestingly, 14B1 potently neutralized BA.2.75 with an EC_50_ value of 41.61 ng/mL, while 14B1 showed no neutralizing activity against the other tested Omicron subvariants. We also evaluated the potential of 7B3 and 14B1 for mAb cocktails and found that the combination of these two mAbs expand the neutralizing breadth against SARS2-CoV-2 variants based on pseudovirus neutralization assay. As these two mAbs partially compete the binding to RBD, we did not pursue the 7B3/14B1 cocktail application further. The authentic SARS-CoV-2 plaque reduction neutralization assay was conducted to corroborate the neutralizing results from the pseudovirus neutralizing assay. Same as VSV pseudovirus results, 7B3 and 14B1 showed potent neutralizing activity against authentic SARS-CoV-2 WT strain and Delta variant and 7B3 also potently inhibits authentic Omicron BA.1 and BA.2 with the EC_50_ values of 259.3 ng/mL and 237.3 ng/mL, respectively (Fig. 3b). 3C4 was the only mAb to neutralize the emerging XBB (EC_50_ = 645 ng/mL) and XBB.1.5 (EC_50_ = 174 ng/mL) variants, even though 3C4 showed no neutralizing activity against all of the other tested Omicron subvariants. This is consistent with our binding data, as only 3C4 displayed a detectable binding signal against XBB-RBD among tested six mAbs, with a kinetically-derived binding affinity of 8.37 nM. However, the binding affinity of the mAbs to the RBDs does not correlate well with the neutralization potency against distinct SARS-COV-2 variants, as 12G4 and 13B2 could bind to Delta-RBD with nanomole to picomole affinities (Supplementary Fig. 2a), respectively, the two mAbs exhibit relatively weaker inhibition activity against Delta strain tested by both pseudovirus and authentic viruses (Fig. 3).

### 7B3 and 14B1 treat SARS-CoV-2 Delta infection in K18-hACE2 KI mice

The Delta variant, once the dominant lineage, has higher infectiousness than the original SARS-CoV-2 and moderate immune escape ability. Considering the potent neutralizing ability of 7B3 and 14B1 against the Delta variant, we evaluated the therapeutic effects of 7B3 and 14B1 in K18-hACE2 KI mice against the SARS-CoV-2 Delta variant; 12 ~ 18-week-old K18-hACE2 KI mice were intranasally infected with 250 PFU/mL Delta variants. One day later, these mice were i.p. injected with 20 mg/kg mAbs followed by daily monitoring.

**Fig. 4:**
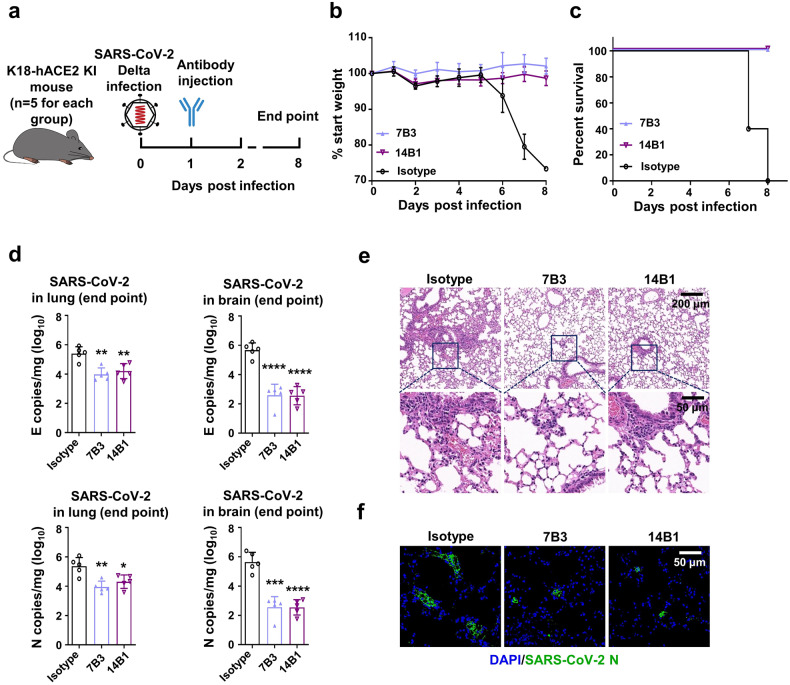
Mabs7B3 and 14B1 treat infection of SARS-CoV-2 Delta variant in vivo. **a** Time schedule for antibody therapy in vivo (*n* = 5 for each group). 12–18-week-old K18-hACE2 mice were intranasally infected with 250 PFU/mL SARS-CoV-2 Delta viruses each at dpi 0, and were injected 20 mg/kg antibodies at dpi 1. Endpoint is at dpi 8 or when mice dead or reach 80% body weight, and the mice were euthanized and samples at endpoint. **b** Body weight changes. **c** Survival rate. **d** The SARS-CoV-2 RNA (E and N gene) copy number in lungs and brains. Unpaired *t* test. *p < 0.05; **p < 0.01; ***p < 0.001; ****p < 0.0001. **e** The H&E stain of lungs. Scar bar, 200 μm in the up row, 50 μm in the down row. **f** The immunofluorescence analysis of SARS-CoV-2 N protein in the lungs. Nuclear DNA is stained by DAPI. Scar bar, 50 μm

As shown in Fig. 4, mice injected with 7B3 or 14B1 survived the infection of the Delta variant, and no significant weight change was observed. However, the isotype control group dramatically lost body weight from 6 dpi, and all mice in this group succumbed to infection by 8 dpi. We also found that 7B3 and 14B1 significantly decreased the viral loads in the lungs and brains compared to the isotype control (Fig. 4a–d). Lung pathology analysis showed that the Delta variant also caused severe interstitial pneumonia characterized by a large number of inflammatory cells infiltrating the lungs and the wide thickening and rupture of the alveolar septum in the isotype group. In contrast, only limited inflammatory cell infiltration was observed in mice injected with 7B3 or 14B1 (Fig. 4e). These results were also consistent with the immunofluorescence analysis, little N protein (green) was detected in the lungs of mice administrated with 7B3 or 14B1 (Fig. 4f).

### 7B3 treats SARS-CoV-2 Omicron BA.1 and BA.2 infections in K18-hACE2 KI mice

The Omicron variant, carrying more than 30 mutations in the S protein, has become the dominant lineage instead of Delta since 2022.^25^ Moreover, Omicron-associated lineages, like BA.1, BA.2, BA.3, BA.4, BA.5, XBB.1.5, XBB.1.16, and XBB.2.3.2, are still emerging. Due to a large number of mutations in the S protein, Omicron has higher infectiousness and stronger immune escape ability than the other SARS-CoV-2 variants, causing many breakthrough infections around the world.

**Fig. 5:**
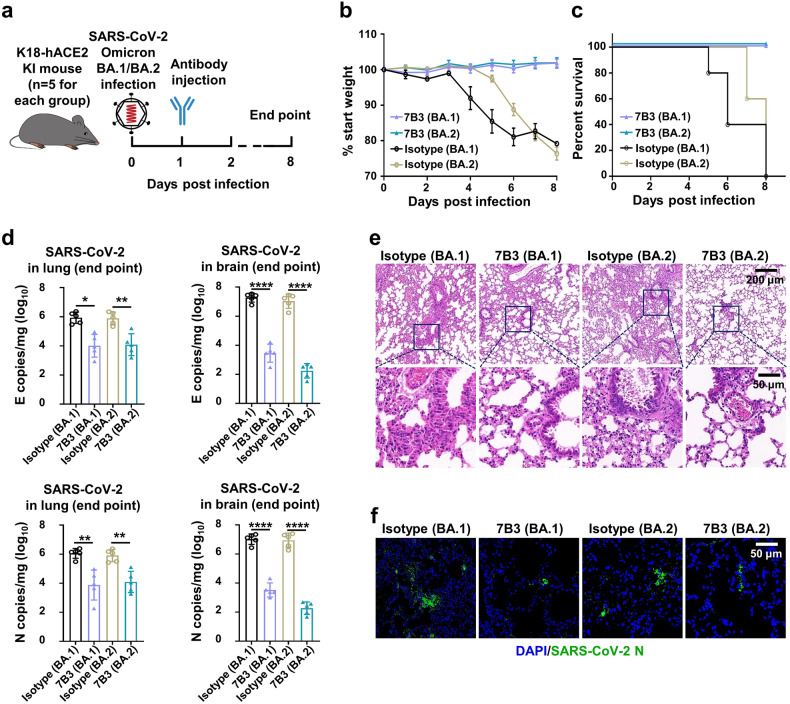
mAbs 7B3 treat infection of SARS-CoV-2 Omicron BA.1 and BA.2 variants in vivo. **a** Time schedule for antibody therapy in vivo (*n* = 5 for each group). 12–18-week-old K18-hACE2 mice were intranasally infected with 500 PFU/mL BA.1 or 2500 PFU/mL BA.2 viruses each at dpi 0, and were injected 20 mg/kg antibodies at dpi 1. Endpoint is at dpi 8 or when mice dead or reach 80% body weight, and the mice were euthanized and samples at endpoint. **b** Body weight changes. **c** Survival rate. **d** The SARS-CoV-2 RNA (E gene and N gene) copy number in lungs and brains. Unpaired *t* test. *p < 0.05; **p < 0.01; ****p < 0.0001. **e** The H&E stain of lungs. Scar bar, 200 μm in the up row, 50 μm in the down row. **f** The immunofluorescence analysis of SARS-CoV-2 N protein in the lungs. Nuclear DNA is stained by DAPI. Scar bar, 50 μm

Though Omicron can largely escape from most neutralizing antibodies, 7B3 was able to neutralize multiple Omicron subvariants including BA.1, BA.2, and BA.3, we then investigated the therapeutic effect of 7B3 in K18-hACE2 KI mice against the Omicron (BA.1 and BA.2) variants. 12 ~ 18-week-old K18-hACE2 KI mice were infected with 500 PFU/mL Omicron BA.1 variant or 2500 PFU/mL Omicron BA.2 on day 0, and then these mice were i.p. injected with 20 mg/kg mAbs on day 1. Encouragingly, mice treated with 7B3 survived the infection of the BA.1 and BA.2 variants, and no significant weight change was observed. However, the isotype control group dramatically lost body weight from 4 dpi for BA.1-challenged mice or 5 dpi for BA.2-challenged mice, and all the control mice succumbed to infection by 8 dpi (Fig. 5b, c). We also found that 7B3 significantly decreased the viral loads in the lungs and brains compared to the isotype control (Fig. 5d). Lung pathology analysis showed that both Omicron BA.1 and BA.2 caused severe interstitial pneumonia characterized by a large number of inflammatory cells infiltrating into the lungs and the wide thickening and rupture of the alveolar septum in the isotype group. In contrast, only limited inflammatory cell infiltration and slight thickening were observed in mice injected with 7B3 (Fig. 5e). The immunofluorescence analysis also showed that less N protein (green) was detected in the lungs of mice treated with 7B3 than control mice (Fig. 5f). These results indicated that 7B3 can efficiently prevent infection by the Omicron subvariants BA.1 and BA.2 in K18-hACE2 KI mice.

### The Cryo-EM structure of 14B1 in complex with SARS-CoV-2 WT Spike

**Fig. 6:**
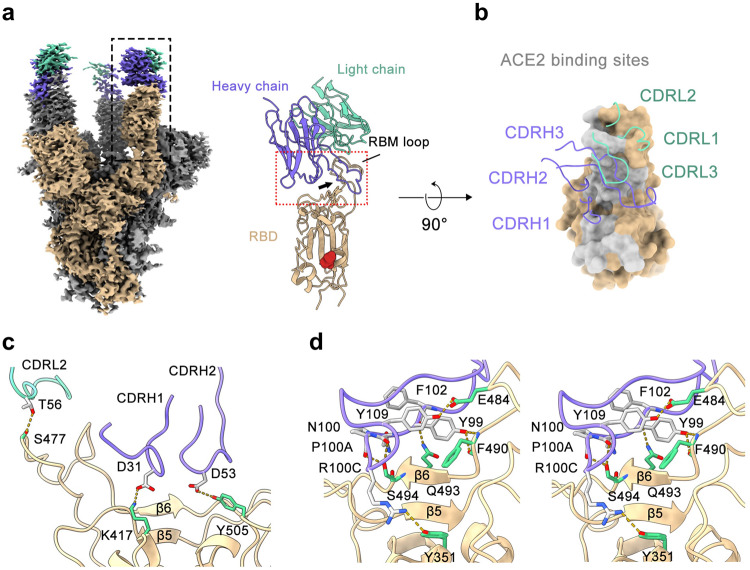
Cryo-EM reconstruction of 14B1-RBD complex. **a** (Left) Cryo-EM structure of three 14B1 Fab in complex with SARS-CoV-2 S-6P trimer. All three Fabs (light chain colored in the green, heavy chain in slate blue) bind on the top face of RBD, with one of three spike protomers colored in beige and the other two in gray. The local refinement region is boxed with black box. (Right) Side view of 14B1 bound RBD after local refinement. The long CDRH3 loop around the RBM loop is indicated by an arrow, and the glycan linked to N343 is marked with red sphere. **b** Top view of 14B1 bound RBD. For simplicity, only the regions with the red dashed box in (**a**) are shown. The CDR loops are labeled, and hACE2 binding motifs (RBM) are highlighted in light gray on the RBD surface colored in beige. **c** Interactions between RBD and the CDRL2, CDRH1, and CDRH2 loops. The residues bound with CDRL2, CDRH1 and CDRH2 are shown as green stick. The color of CDR loops is similar to (**b**) and the residues bound with RBD are shown as gray stick. The yellow dashes represent hydrogen bonds and salt bridges. **d** Stereo pairs showing interactions between CDRH3 and RBD. The color scheme is similar to (**c**)

To understand the 14B1-mediated neutralization mechanism, we obtained cryo-electron microscopy (cryo-EM) reconstruction of 14B1 Fab in complex with the ectodomain of the trimeric spike protein (WT-Spike-6P) at an overall resolution of 3.2 Å. Only one Spike state was identified, with three up-RBDs occupied by three 14B1 Fabs (Fig. 6a). To obtain more detailed information on antibody-antigen interactions, we focused on the RBD-variable fragment (Fv) of 14B1 and generated a local refined map (Supplementary Fig. 4). Structural analysis shows that 14B1 resides on the top of RBD, and the binding site overlaps partially with binding residues made by hACE2 (Fig. 6b), belonging to the RBD-2 antibody in the Hastie’s classification system.^19^

The 14B1 paratope buries a surface area of ~1055 Å^2^ on one up-RBD, calculated by PISA,^26^ and the heavy chain accounts for ~80%. The complementarity-determining regions of the heavy chain (CDRHs) straddle the “RBD ridge”^4^ (Fig. 6b) to dominate the 14B1-RBD interaction. In contrast, the light chain of the antibody shows less contact with the RBD, and mainly interact with RBD through one hydrogen bonds formed between Thr^56^ and Ser^477^ (Fig. 6c). CDRH1 and CDRH2 bind to an epitope on the RBD that faces the threefold axis of the spike, which is inaccessible in the RBD-down conformation. Asp^31^ from CDRH1 and Asp^53^ from CDRH2 form hydrogen bonds with Lys^417^ and Tyr^505^, respectively (Fig. 6c and Supplementary Table 2). Notably, the 26-residue-long CDRH3 loop (Supplementary Fig. 7) kinks at Ala^93^ and Ile^102^ to form a hook-like loop span the saddle (β5-β6) of RBD and plays a crucial role in the Fab-RBD association, accounting for 74% (645.6 Å^2^) of the heavy chain paratope. CDRH3 comprises a series of aromatic and hydrophobic residues at the center of the paratope and forms multiple hydrophobic interactions and hydrogen bonds with RBD, including hydrogen contacts formed between Phe^98^, Tyr^99^, Asn^100^, Pro^100A^, Arg^100C^, and Tyr^100E^ of CDRH3 and Gln^493^, Phe^490^, Ser^494^, Try^351^, and Glu^484^ of RBD, respectively (Fig. 6d and Supplementary Table 2).

### The Cryo-EM structure of 7B3 in complex with SARS-CoV-2 WT spike

**Fig. 7:**
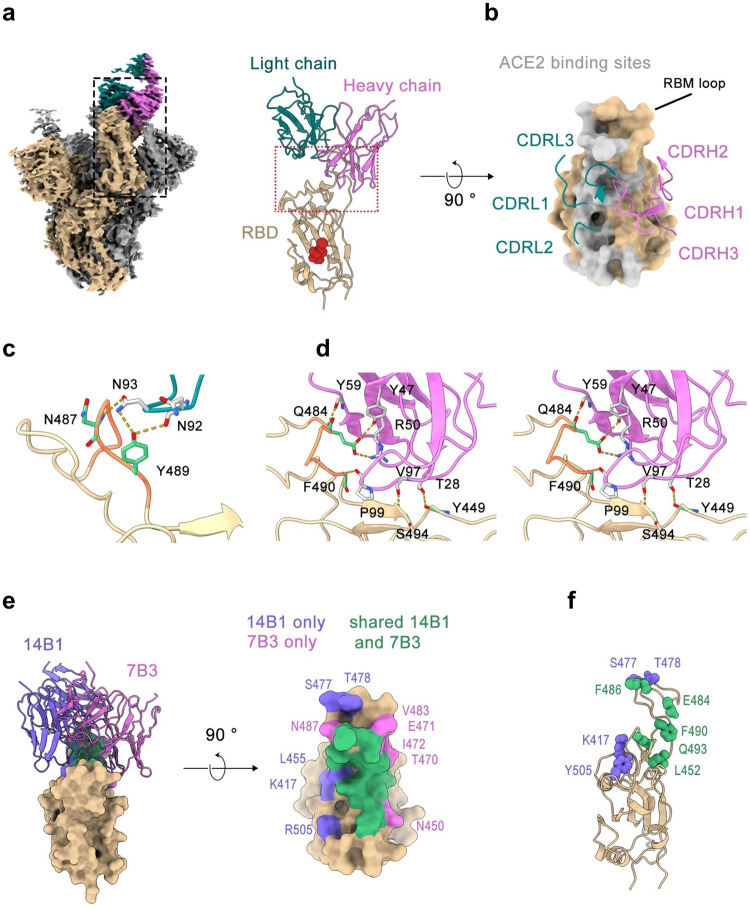
Cryo-EM reconstruction of 7B3-RBD complex and structure superposition. **a** (Left)The overall structure of 7B3 Fab in complex with SARS-CoV-2 S-6P trimer. One Fab (light chain in teal, heavy chain in pink) bounded RBD is in the “down” position, and the Fab bound spike protomer colored in beige and the other two in gray, and the local refinement region is boxed with black dash. (Right) Side view of 14B1 bound to RBD model after local refinement, and the N343 linked glycan is shown as red sphere. **b** Top view of 7B3 bound RBD. The CDR loops are labeled, RBD is shown as beige surface, and hACE2 binding sites are colored in light gray. **c** Interactions between RBD and CDRL3 loop. The residues bound with CDRL3 are shown in green stick. CDRL3 are in teal and the residues bound with RBD are shown in gray stick models. The yellow dashes represent hydrogen bonds and salt bridges. Residues 483–490 (VEGFNCYF) are colored in coral. **d** Stereo pairs showing interactions between heavy chain and RBD. The color scheme is similar to (**c**). **e** Structure superposition of 14B1-RBD and 7B3-RBD. (Left) 14B1 (slate blue ribbon model) and 7B3 (pink ribbon model) both bind to the top face of RBD. (Right) Top view of the epitopes of 14B1 and 7B3, and the epitopes of two Fabs are partially overlapped. Epitopes unique to the two antibodies are labeled separately. **f** Mutated residues contacted by 14B1 or 7B3 on SARS-CoV-2 WT RBD in comparison with Omicron variants are shown as spheres. Color scheme is similar to (**e**)

We also determined the structures of 7B3 Fab in complex with the SARS-CoV-2 WT-Spike-6P and Omicron BA.1-Spike-6P (Omicron-Spike-6P) at an overall resolution of 3.5 Å and 3.4 Å, respectively, and found that 7B3 showed identical binding mode to both WT-Spike-6P (Fig. 7) and Omicron-Spike-6P (Supplementary Fig. 6). Due to the lack of details for the locally refined 7B3-Omicron-Spike-6P complex map (Supplementary Fig. 6e), we then use a relatively higher resolution complex structure of 7B3-WT-spike-6P for further analysis. Unlike 14B1, 7B3 Fab can bind RBD with both “up” and “down” conformations on the Spike (Supplementary Fig. 5), although the epitope of 7B3 on Spike is partially overlapped with 14B1 binding sites (Fig. 7e). The RBD conformation of some models generated in the 3D classification procedure can be identified with two “up” and one “down”, but only the “down” RBD region is sufficient for local refinement.

Local refinement was performed and improved the local resolution of the 7B3-RBD interface to 3.9 Å (Supplementary Fig. 5). 7B3 bind at the straddle (β5–β6) and is closely associated with the RBM loop (Fig. 7a). The total buried surface area is 1023 Å^2^ from the RBD, and the paratope constituted all six CDRs, with the heavy chain and light chain contributing 73 and 27% of the binding surface area, respectively (Fig. 7b and Supplementary Table 3). The medium-resolution complex structure revealed that residues 483–490 (VEGFNCYF) located at the RBM loop predominantly contribute to the 7B3-RBD interaction by forming extensive hydrogen bonds made by residues Glu^484^, Asn^487^, Tyr^489^, Phe^490^ with Tyr^47^, Tyr^59^ in or adjacent to CDRH2, Asn^92^, Asn^93^ in CDRL3 and Pro^99^ in CDRH3 of 7B3, respectively (Fig. 7c, d). The interaction of 7B3 to RBD is mediated by eight hydrogen bonds and one salt bridge formed between Glu^484^ in RBD and Arg^50^ in CDRH2 of the heavy chain, in addition to van der Waals contacts. By superposing the RBD region of antibodies-RBD models, 7B3 would clash with 14B1 (Fig. 7e), suggesting that the two mAbs bind competitively, which is consistent with the competition BLI results.

### The molecular basis of SARS-CoV-2 variants neutralization by 14B1 and 7B3

Although 7B3 partially affects 14B1’s interaction with RBD by competition BLI, the complex structures showed that 7B3’s epitope is not completely overlapped with 14B1’s binding footprint, and the proximal contacts made by 14B1 and 7B3 are distinct (Fig. 7e). Structural analysis showed that 14B1 recognized the peak of the RBM and sterically blocked the hACE2 binding to the RBD. Heavy-chain complementarity-determining regions (CDRs) contribute the majority of interactions with RBD, comprising ten hydrogen bonds targeting several residues between 484–505 and the residues Y351 and K417 of RBD. 14B1 efficiently neutralized WT as well as Alpha (B.1.1.7), Beta (B.1.351), Gamma (P.1), Delta (B.1.617.2), and Eta (B.1.525) but exhibited diminished inhibitory activity against most Omicron subvariants. Among 17 interface residues, Eta (B.1.525) carrying E^484^K mutation, Beta (B.1.351) and Gamma (P.1) carrying K417N/T and E484K mutations, and Delta carrying L^452^R/T^478^K mutations, while L^452^ and T^478^ only contribute 1 and 2 van der Waals contacts to bind to S^100B^ (CDRH3) and H^49^ (CDRH2), respectively. Although K^417^ and E^484^ engaged in hydrogen bonds interaction with the heavy chain of 14B1, these two mutations slightly affect the binding capacity of 14B1 to the RBD (Supplementary Fig. 2), indicating that these two amino acids play minor roles in the binding and neutralization of 14B1.

There are more than 15 mutations in the RBD of the Omicron variants compared to the ancestral SARS-CoV-2 strain, 9 and 5 of which reside in the interface of 14B1 and 7B3, respectively (Fig. 7f). Based on amino acids sequence alignment and structural analysis, we found that K^417^, Y^505^, and Q^493^ of RBD formed hydron bonds with CDRH3 of 14B1 and were involved in extensive interaction between 14B1 and WT-Spike-6P, the substitution of K^417^N, Y^505^H, and Q^493^R in most of Omicron VOAs profoundly disrupted or reduced the binding of Omicron-Spike with 14B1 (Supplementary Fig. 9), which likely were responsible for the 14B1 neutralization resistance against most Omicron variants. Among the three contacts, residues 493 possibly play the most dominant role as Omicron variant BA.2.75 harboring Q on residue 493 became sensitive to 14B1 neutralization, and only this residue difference on the interface were observed between Omicron variant BA.2.75 and resistant variants Omicron BA.1, BA.2 and BA.3.

7B3 predominantly interacts with SARS-CoV-2 WT-spike at regions 450–457,470–472, and 483–494 using both heavy and light chains, and the contacts made by 7B3 on WT exhibited 100 and 94% amino acid sequence identity with Alpha (B.1.1.7) and Delta (B.1.617.2), respectively, which correspond to its neutralization breadth. Interestingly, 7B3 also potentially inhibits highly mutated Omicrons, including Deltacron, Omicron BA.1, BA.2, BA.3, and BA2.75, and shows weak or little neutralizing activity against other Omicron variants. The mutant residues of E^484^A in Omicron BA2.75 and E^484^A combined with Q^493^R in Omicron BA.1, BA.2, and BA.3 are located in the interface between the 7B3 and RBD. Although Omicron BA.1-RBD carrying E^484^A and Q^493^R showed reduced binding affinity to 7B3 than WT-RBD, it still could bind to 7B3 very well with a kinetic-derived binding affinity of 14.22 nM (Supplementary Fig. 2). Structurally, Q^493^ makes slight contact with 7B3, and E^484^ forms two hydrogen bonds and several van der Waals contacts with heavy and light chains of 7B3 (Supplementary Tables 2 and 3). It looks like E^484^ was essential for its neutralization activity as 7B3 lost the inhibition activity against Beta and Gamma variants, and only one mutation (E^484^K) was present in the interface of 7B3 with Beta- and Gamma-Spike. However, the E to A mutation in residues of 484 in Omicron BA.1, BA.2, and BA.3 was adorable and still sensitive to 7B3’s neutralization.

## Discussion

In this study, we developed a panel of human mAbs from the PBMC of two convalescents infected by the original SARS-CoV-2 strain and characterized these mAbs structurally and functionally. We found that the mAbs exhibited distinct inhibitory activity against different SARS-CoV-2 VOCs. Of these, the potent mAb 3C4 was able to inhibit the emerging Omicron XBB variants infection with intermediate neutralizing potency. The highly potent mAbs, 7B3 and 14B1, showed broad neutralizing activities against SARS-CoV-2 and bound the peak of the RBM from different angles. These two mAbs protected K18-hACE2 mice from lethal viral infection in the face of SARS-CoV-2 WT or Delta (B.1.617.2) challenge, and 7B3 also showed therapeutic activity against lethal Omicron infection in vivo.

Although numerous mAbs targeting similar epitopes have been reported, most of them are derived from frequently used mAb heavy chain genes. mAb 14B1 utilizes the VH3-43 germline sequence, and so far, only two VH3-43 derived mAbs (GW01, S2K146) complexed with SARS-CoV-2 RBD/Spike are available in the PDB.^27,28^ Although they derived from the same heavy-chain variable region, GW01 targeted the inner face of RBD and was grouped into the RBD-7 community. 14B1 and S2K146 use distinct light chain genes, while the two mAbs target similar antigenic sites on RBD primarily mediated by heavy chain. 14B1 has a relatively longer CDRH3 loop with 26 amino acids due to a large number of amino acids insertion at the junctions of VDJ rearrangements, which makes the primary contacts with RBD (Supplementary Fig. 7). The other two heavy chain residues involved in the RBD interaction are D31 from CDR1 and D53 from CDRH2, and these two residues use the germline sequences to contact RBD. Similar to 14B1, S2K146 has a 15-amino-acid-long CDRH3 loop that contributes to the majority of the interactions with RBD. Among the 12 contact residues, only one somatic mutant was observed for mAb S2K146, and no somatic mutant residues from the heavy chain are involved in the interaction with RBD. The results indicate that VH3-43 germline-like mAbs may be capable of neutralizing SARS-COV-2 infection with great breadth and potency, although more VH3-43-derived RBD mAbs need to investigate.

VH1-69 is a widely used antibody gene against SARS-CoV-2 infection, and VH1-69-encoded mAbs target multiple regions of the Spike, including a subset of epitopes on RBD and NTD.^21,29,30^ Although most VH1-69-encoding RBD-binding mAbs lost neutralizing activity against Delta due to the substitution of residue L452 with R, they also showed weak or no capability of neutralization against emerging Omicron strains.^29^ mAb 7B3, encoded by VH1-69, potently neutralized multiple SARS-CoV-2 pseudoviruses (WT-D614G, Alpha, Delta, Deltacron, and Omicron BA.1, BA.2, BA.3 and BA2.75), and it also showed comparable inhibitory activity against WT, Delta and Omicron BA.1, BA.2 authentic viruses. In a lethal viral infection mouse model, 7B3 showed prophylactic efficacy against WT challenge and therapeutic efficacy against Delta and Omicron BA.1 infection. Our finding suggests that public mAb 7B3 is a promising candidate for antibody therapeutics against SARS-CoV-2 infections. We acknowledged that the influence of different routes of administration should be considered when evaluating the efficacy of mAbs in vivo and the different mAb delivery routes comparison warrant further investigation.

14B1 and 7B3, derived from one convalescent, provided potent protection against infections by SARS-CoV-2 WT and multiple variants in vitro and in vivo, including WT, Alpha, Beta, Gamma, Delta, and multiple Omicron subvariants (BA.1, BA.2, BA.3 and BA2.75). Omicron subvariant XBB and its progeny (XBB.1, XBB.1.5) caused another wave of infection around the world and rendered all authorized monoclonal antibodies inactive.^31–34^ One of our mAb, 3C4, still effectively neutralized the XBB and XBB.1.5 variants, with EC_50_ values of 645 ng/mL and 174 ng/mL, respectively, even though 3C4 was derived from the earliest COVID-19 convalescents who were infected by the original Wuhan strain. These results indicate that original SARS-CoV-2 infection or vaccination can activate broad humoral immunity, can induce diverse antibodies with different neutralizing profiles, and may help people fight against the emerging Omicron and potential future variant pandemics.

In summary, many studies have reported that serum samples from individuals recovered from SARS-CoV-2 infection or vaccinees showed a reduced neutralizing potency against SARS-CoV-2 VOCs. We found that even though a significant immune evasion was observed at the serum level, mAbs with broadly neutralizing activity against VOCs can be isolated from subjects infected with the original strain. Two highly potent mAbs recognized RBM from different directions and effectively protected mice from lethal SARS-CoV-2 WT, Delta, and even Omicron challenge. This study expands the extent of the anti-SARS-COV-2 mAbs library and provides important insights into humoral immunity and protection against SARS-CoV-2 infection.

## Materials and methods

### Sample collection and processing

Two eligible individuals were recruited according to our previous study,^6^ and their blood samples were collected in January 2021. The sample collection was applied by the Hubei Provincial Center For Disease Control and Prevention and Hubei Provincial Academy Of Preventive Medicine (HBCDC) with written consent under appropriate institutional review Boards approval (2021-012-01) and was deidentified. PBMCs were separated from these blood samples via the Human Lymphocyte Separation Tube (DAKEWE, 7121011) by following the manufacturer’s instructions. After the lysis of red blood cells (Biolegend, 420301), these PBMC samples were stored in liquid nitrogen.

### Staining and flow cytometry

PBMCs from COVID-19 convalescents were stained by FVS-780, CD3-BV510, CD4-BV510, CD8-BV510, CD19-PE, IgD-BB700, CD20-BV421, and S1-Biotin for 30 min at 4 °C. After washing, Streptavidin-APC was added to these PBMCs for 30 min at 4 °C. These samples were then washed and loaded for flow cytometry. The gating strategy was CD19^+^, CD3^−^, CD4^−^, CD8^−^, IgD^−^, CD20^+^, and S1^+^. The target cells were sorted into 96-well plates and stored at −80 °C.

### Sequencing and clone construction

Following the protocol from ref. ^35^ reverse transcription, nested, and cloning PCRs were applied to obtain the heavy- and light-chain variable regions of the antibodies. The paired heavy- and light-chain genes were codon-optimized, synthesized, and cloned into expression vectors containing the human IgG_1_ constant regions.

### Expression and purification of mAbs

HEK293F cells were cultured in SMM 293T-I medium (Sino Biological) at 37 °C, with 5% CO_2_. Equal amounts of paired heavy- and light-chain plasmids of antibodies were transfected into HEK293F when the cell density is 1 × 10^6^ cells per mL with EZ Trans (Life-Lilab) transfection reagent. The cell supernatant was collected by centrifugation to remove the cells, and Protein A Resin (GenScript) was used to purify the mAbs. These mAbs were further purified by a Superose 6 10/300 column (GE Life Sciences) and resuspended into PBS, and the purity of the recombinant antibodies was analyzed by SDS-PAGE.

### ELISA

The ELISA experiments were conducted to measure the binding ability of mAbs against the SARS-CoV-2 WT, Delta, BA.1, and XBB.1.5 RBD protein. Briefly, serially diluted mAbs were added to 96-well plates precoated with RBD. Then, the plates were incubated at 37 °C for 2 h, followed by 5-times washes. After that, goat anti-human IgG antibodies (AP-conjugated, Southern Biotech) were added and incubated at 37 °C for 1 h. At last, the substrate 4-nitrophenyl phosphate disodium salt hexahydrate (Sigma) was added to the plates, and 45 min later, the absorbance at 405 nm was measured by an ELISA plate reader (Tecan).

### Biolayer interferometry binding assay

The binding affinity of the purified SARS-CoV-2 Spike protein or RBD protein with mAbs was monitored by BLI using an Octet-Red96 device (Pall ForteBio). Briefly, 20 μg/ml mAbs were loaded onto protein A (ProA) biosensors (ForteBio, 18–5010), and then dipped into PBST buffer to wash out unbound mAb. Then, the biosensors were dipped into PBST buffer containing soluble Spike protein or RBD protein with concentrations ranging from 0 to 300 nM and then dipped into PBST buffer to record dissociation kinetics. PBST buffer was used to define the background. The affinities were calculated by Octet Data Analysis software.

### Epitope competition assay

The competition between mAbs and hACE2 was detected by BLI using an Octet-Red96 device (Pall ForteBio). Briefly, 20 μg/ml mAbs was loaded onto protein A (ProA) biosensors (ForteBio, 18–5010). The loaded biosensors were then dipped into the kinetic buffer (PBST) to wash out unbound mAb. Subsequently, the biosensors were dipped into the kinetic buffer containing 200 nM RBD and then dipped into kinetics buffer containing hACE2 or other mAbs for the epitope competition test. Kinetic buffer was used to define the background.

### SARS-CoV-2 pseudovirus production and titration

SARS-CoV-2 spike protein pseudotyped virus (SARS-CoV-2-psV) was producted based on a previously described protocol using a replicate-deficient VSV-based rhabdoviral pseudotyping system (VSV-dG) as we mentioned before.^6,36^ And the TCID_50_ of the pseudovirus was determined using a serial dilution-based infection assay on BHK-21-hACE2 cells and calculated according to the Reed–Muench method.^37,38^

### SARS-CoV-2 pseudoviruses neutralization assay

Serial dilutions of antibodies were mixed with pseudoviruses (2 × 10^3^ TCID_50_/well), incubated for 30 min at 37 °C, and then added to BHK-21-hACE2 cells at a density of 2 × 10^4^/well in a 96-well plate. 16 h later, cells were lysed by 1 × passive lysis buffer (Promega, E1941) at room temperature for 15 min. Luciferase activity in the cell lysate was determined by a Bright-Glo luciferase assay kit (Promega) and measured through a GloMax® 20/20 Luminometer (Promega).

### Authentic SARS-CoV-2 WT and variants neutralization assay

The SARS-CoV-2 WT strain (IVCAS 6.7512) was provided by the National Virus Resource, Wuhan Institute of Virology, Chinese Academy of Sciences. The B.1.617.2 strain (YJ20210707-01), BA.1 strain (YJ20220223), and BA.2 strain (YJ20220310) were provided by Hubei Provincial Center for Disease Control and Prevention. All SARS-CoV-2 authentic virus-related experiments (S01321010A) were approved by the Biosafety Committee Level 3 (ABSL-3) of Wuhan University. In brief, mAbs or sera were serially diluted in a culture medium and mixed with 100 PFU SARS-CoV-2 for 30 min at room temperature. The mixture was then added to Vero E6 cells in 24-well plates and incubated for 2 h, and the supernatant was replaced by DMEM with 5% FBS and 1% methylcellulose. Three days later, the supernatant was discarded and the cells were fixated with 4% paraformaldehyde, followed by the stain of crystal violet. The plaques were counted and analyzed by GraphPad Prism 7 software to calculate the 50% effective concentration (EC_50_) of antibodies.

### Antibody protection and treatment of K18-hACE2 KI mice against SARS-CoV-2 infection

K18-hACE2 KI mice, which express hACE2 driven by the human epithelial cell cytokeratin-18 (K18) promoter, were purchased from Gempharmatech. All animal experiments were approved by the Animal Care and Use Committee of Wuhan University. For antibody prophylactic experiments, mice were grouped for injection of antibodies (10 mg/kg). At dpi 0, mice were inoculated intranasally with 250 PFU SARS-CoV-2. For antibody therapeutic effect, mice were inoculated with 250 PFU of SARS-CoV-2 by the intranasal route, and one day later, mice were injected with antibodies (20 mg/kg). Body weights were monitored daily, and the endpoint is at dpi 8 or when mice dead or when mice’s weight reached 80% of starting weight. Tissues were harvested for tissue homogenates and histology analysis. The H&E and immunofluorescence stain and imaging were carried by Wuhan Pinuofei Biological Technology company. The viral gene copies were detected by a TaqMan RT-PCR Kit (Yeason). To accurately quantify the absolute number of SARS-CoV-2 genomes, a standard curve was prepared by measuring the SARS-CoV-2 E and N gene plasmid.^39^ All animal studies were approved by the Institutional Animal Care and Use Committee at Wuhan University (WP20220044).

### Protein production and purification

DNA fragment encoding ectodomain of S from SARS-CoV-2 with the disruption of S1/S2 furin cleavage site and the substitutions of six amino acids with proline (F817P, A892P, A899P, A942P, K986 P, V987P) was placed into the mammalian expression vector pCAGGS with N-terminal signal peptide (S-6P). The C terminus was engineered with a thrombin site (LVPRGS) linked by a folded trimerization motif (YIPEAPRDGQAYVRKDGEWVLLSTFL) and a 6×His Tag. The S-6P plasmid was transiently transfected into Expi293 cells using PEI reagent. The cell supernatants containing spike protein were harvested 108 h after transfection, clarified by centrifuge (20 min at 8000 × *g*, 4 °C), and filtered through a 0.45 μm filter. The soluble S proteins were recovered using 2 ml of High-Affinity Ni-charged Resin (GenScript), further purified by passage over Superose^TM^ 6 Increase 10/300GL (Cytiva).

Variable regions of heavy and light chains were cloned into a Fab expression vector and sequence-corrected plasmids were transiently co-transfected into Expi293 cells with a ratio of 1:1. At 144 h post-transfection, cell supernatants were collected and secreted Fabs were purified with High-Affinity Ni-charged Resin (GenScript), following the purification by gel filtration of Superose^TM^ 200 Increase.

### Cryo-EM sample preparation and data collection

Fabs of 14B1 or 7B3 was mixed with the prefusion-stabilized spike ectodomain trimer (WT-Spike-6P and Omicron-Spike-6P) in a molar ratio of 3.6:1 and diluted to a final concentration of 0.8 mg/ml with the buffer containing 50 mM Tris-HCl pH 8, 150 mM NaCl. After 30 min incubation on ice, 3 μl of the mixture was loaded onto a glow-discharged holy-carbon grid (QuantiFoil, R1.2/1.3). The grid was plunge-frozen into liquid ethane using Vitrobot Mark IV (ThermoFisher Scientific) after blotting under 100% humidity, 16 °C for 3.5 s. Vitrified grids were loaded into a CRYO ARM 300 electron microscope equipped with a Gatan K3 direct electron detector (Table S1). Datasets were collected with serialEM^40^ at a nominal magnification of 50,000× using a defocus range of 0.5 to 2.5 μm in the super-resolution mode, corresponding to a pixel size of 0.475 Å. Each movie stack was dose-fractionated to 40 frames with a total electron exposure of ~40 e-/ Å^2^.

### Cryo-EM data processing

The movie stacks were then 2 × 2 binned and motion-corrected using MotionCor2^41^ implemented in RELION.^42^ The dose-weighted images were imported into cryoSPARC^43^ for the following image processing steps.

After CTF estimation and exposure curation, micrographs were manually selected. Particles were picked and extracted with a box size of 512 × 512 (Fourier cropped to the box size of 256 × 256) (Supplementary Figs. 4 and 5). After reference-free 2D classification and heterogeneous refinement, particles were re-extracted with box size 384 × 384. One more round of heterogeneous refinement was performed and the global resolution was 3.2 Å, 3.5 Å and 3.4 Å for the map of the 14B1-, 7B3- bound WT-Spike-6P and 7B3-bound Omicron-Spike-6P. Local refinement was performed to better solve the electron density of the Fab-RBD interface. For excluding the effects of excess electron density, particles were subtracted with a mask covering the core region of the spike. Based on the pseudo-C3 symmetry of the 14B1-WT-Spike-6P complex, symmetry expansion (C3 point group) was applied to increase the effective number of particles. A mask encompassing RBD and the variable region of antibodies was used for refinement, yielding a local density map at 3.6 Å for 14B1-RBD, 3.9 Å for 7B3-RBD(WT) and 4.3 Å for 7B3-RBD (Omicron BA.1).

### Cryo-EM model building and refinement

The cryo-EM structure of SARS-CoV-2 RBD complexed with P2B-2F6 Fab (PDB ID: 7BWJ) was used as the starting model for model building. Each chain was docked into the density map using UCSF ChimeraX^44^ followed by iterative rounds of manual model adjustment in COOT^45^ and real space refinement with PHENIX.^46^ Structural validation was performed using Molprobity.^47^ Buried surface areas were calculated using PDBePISA v1.52^26^ with a probe of 1.4 Å. Hydrogen bonds were identified using LigPlot+ suite with a cutoff distance of 3.9 Å. Figures were prepared using ChimeraX.^44^

### Statistical analysis

Contrast and image sizes of images were adjusted with Adobe Photoshop. Graphs were produced in GraphPad Prism. All Figures were made in Adobe Illustrator. Sample size is indicated on the corresponding graph or figure legend. Error bars represent the SEM or SD as indicated in the figure legends. Unpaired *t* test was used.

### Supplementary information

Supplementary Materials

## Data Availability

The atomic models and local cryo-EM maps generated of the 14B1-RBD, 7B3-RBD complex (PDB 8I3U,8I3S; EMD EMD-35156, EMD-35155) can be found at the Protein Data Bank (PDB) and Electron Microscopy Data Bank.
