# Supplementary material for: Broadly neutralizing antibodies derived from the earliest COVID-19 convalescents protect mice from SARS-CoV-2 variants challenge
Source: Signal Transduct Target Ther. 2023 Sep 14;8:347. doi: 10.1038/s41392-023-01615-0 (PMC10499932; doi:10.1038/s41392-023-01615-0)
Supplement: Supplementary file 1 — Supplementary Materials [file 41392_2023_1615_MOESM1_ESM.docx]

Supplementary Materials for

Broadly neutralizing antibodies derived from the earliest COVID-19 convalescents protect mice from SARS-CoV-2 WT and VOC variants challenge

Qianyun Liu^†^, Haiyan Zhao^†^, Zhiqiang Li^†^, Zhen Zhang^†^, Rui Huang^†^, Mengxue Gu, Ke Zhuang, Qing Xiong, Xianying Chen, Weiyi Yu, Shengnan Qian, Yuzhen Zhang, Xue Tan, Muyi Zhang, Feiyang Yu, Ming Guo, Zhixiang Huang, Xin Wang, Wenjie Xiang, Bihao Wu, Fanghua Mei, Kun Cai, Limin Zhou, Li Zhou, Ying Wu^*^, Huan Yan^*^, Sheng Cao^*^, Ke Lan^*^, Yu Chen^*^

Correspondence to: Yu Chen, [chenyu@whu.edu.cn](mailto:chenyu@whu.edu.cn); Ke Lan, [klan@whu.edu.cn](mailto:klan@whu.edu.cn); Sheng Cao, [caosheng@wh.iov.cn](mailto:caosheng@wh.iov.cn); Huan Yan, [huanyan@whu.edu.cn](mailto:huanyan@whu.edu.cn); Ying Wu, [yingwu@whu.edu.cn](mailto:yingwu@whu.edu.cn).

**This PDF file includes:**

Figures. S1 to S9

Tables S1 to S3


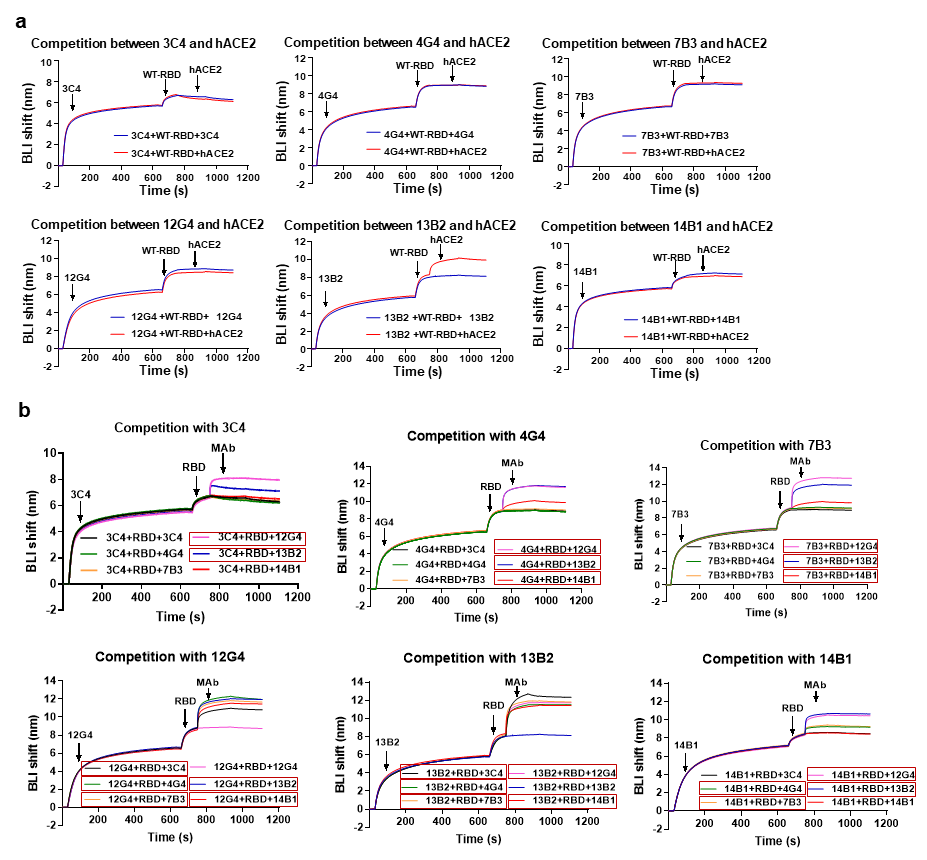


Figure. S1.

The epitope competition between mAbs and hACE2. The mAbs (indicated by black arrows at left in each graph) were loaded onto protein A biosensors followed by the association with the WT-RBD (Second arrow). The hACE2 (**a**) or tested mAbs (**b**) were then loaded to detect the competition relationships of the first loaded mAb with hACE2 of second mAb (Third arrow). The red box indicated the mAbs with no or partial competition. The BLI traces are representative of three independent experiments.


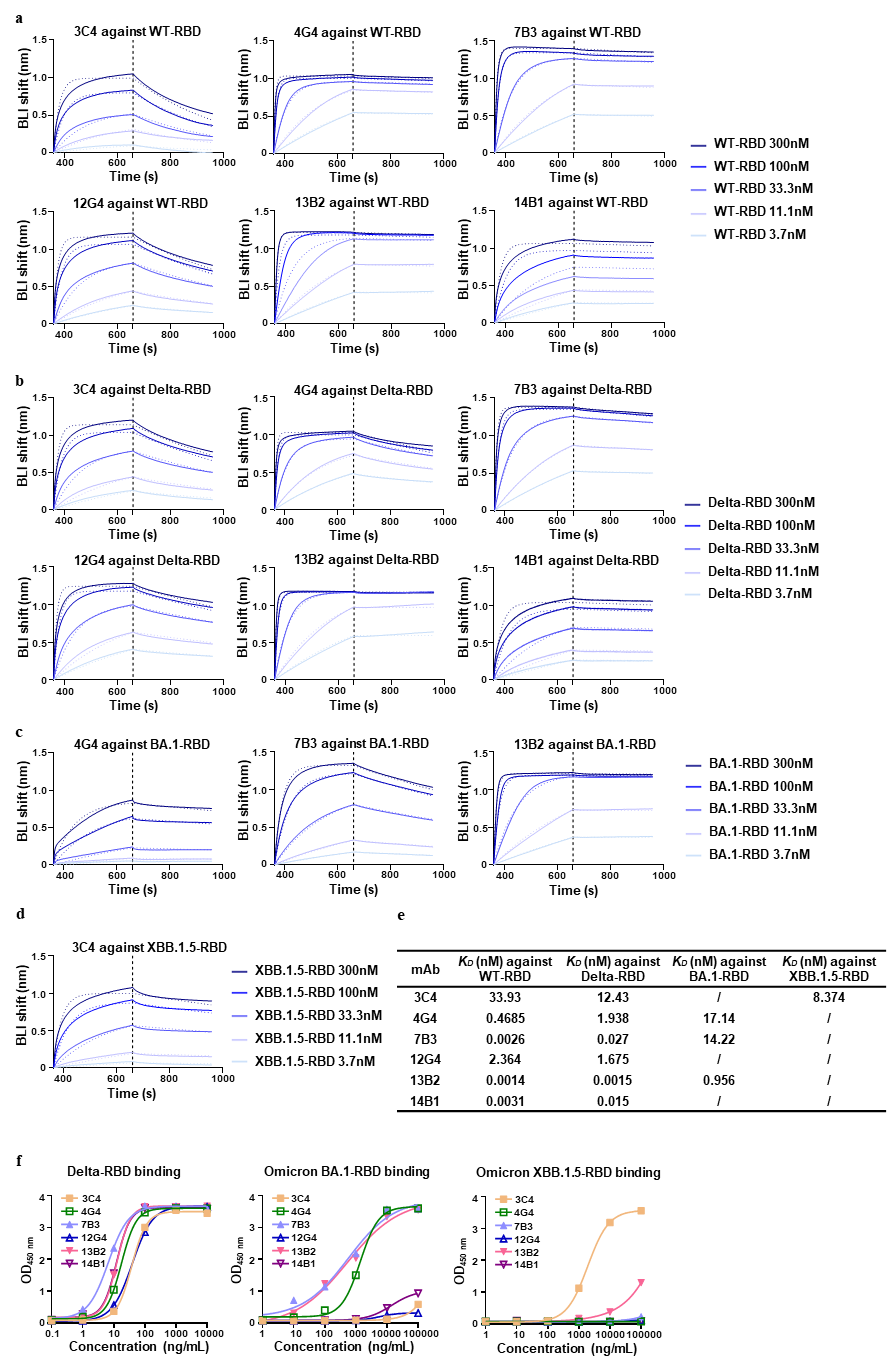


Figure. S2.

The binding capacity of mAbs against RBD of SARS-CoV-2 variants through BLI and ELISA. The affinity of mAbs for WT-RBD (**a**), Delta-RBD (**b**), BA.1-RBD (**c**), and XBB.1.5-RBD (**d**) has been detected by BLI. **e** The summary of the kinetic-derived binding affinity (*K_D_*). /, no association detected. The binding activity of mAbs against Delta-RBD, BA.1 RBD, and XBB.1.5 RBD has also been evaluated by ELISA (**f**). The results are averaged from two independent experiments performed in triplicate.


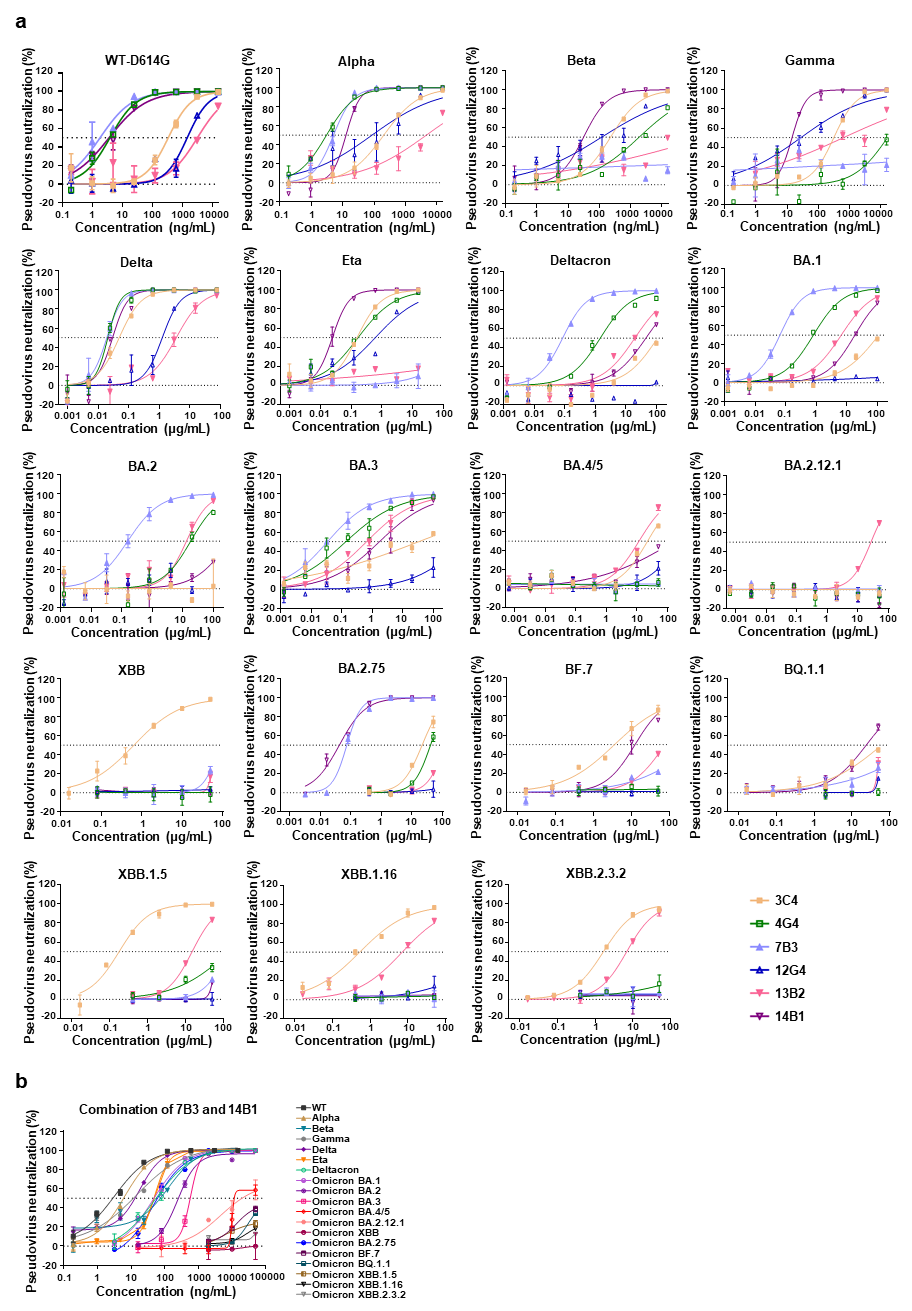


Figure. S3.

SARS-CoV-2 pseudovirus neutralizing assay for mAbs (**a**) and mAb cocktail (**b**). The indicated mAbs were serially diluted and then were inoculated with 3000 TCID_50_ pseudoviruses as indicated for 30 min at room temperature. BHK-21-hACE2 cells were infected by this mixture, followed by a chemiluminescence detection after 16 h. The NT_50_ was calculated by GraphPad Prism 7 software with nonlinear regression curve fitting (normalized response, variable slope). The horizontal dotted lines on each graph indicate 50% and 0% neutralization.


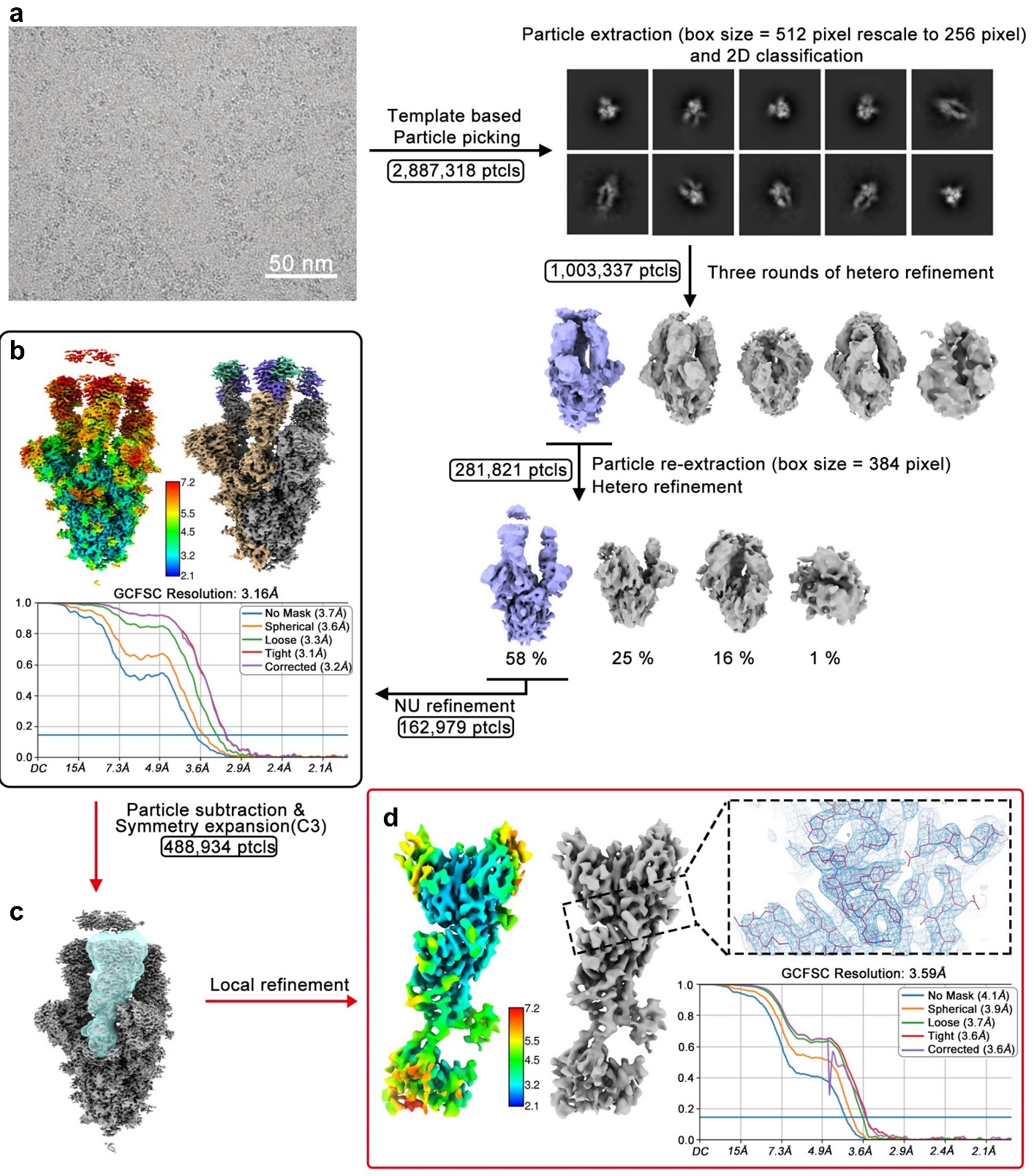


Figure. S4.

Flowchart depicting the cryo-EM data processing steps for the 14B1-spike whole map and 14B1/RBD locally refined map. **a** After 2D classification, classes with distinctive features were selected for subsequent 3D classification. Class colored purple was used for further classification, a pseudo C3 class with the most particles and more completeness was selected. **b** NU refinement with C1 symmetry was performed and generated a map with an overall resolution of 3.2 Å and a local resolution about 7 Å in the region of the RBD and 14B1 interaction. **c** Local refinement strategy was adopted for further improving the resolution, particle subtraction was performed to subtract the density in the core region of spike and symmetry expansion procedure with C3 symmetry was used to increase effective particles. A mask colored with transparent cyan that encompassing one RBD and mAb density was created to define the region for local refinement. **d** Two rounds of local refinement were performed with optimized “soft padding” parameter to help the program find correct orientation of the particles. A zoomed-in view of 14B1 binding region and the FSC curves were shown aside.


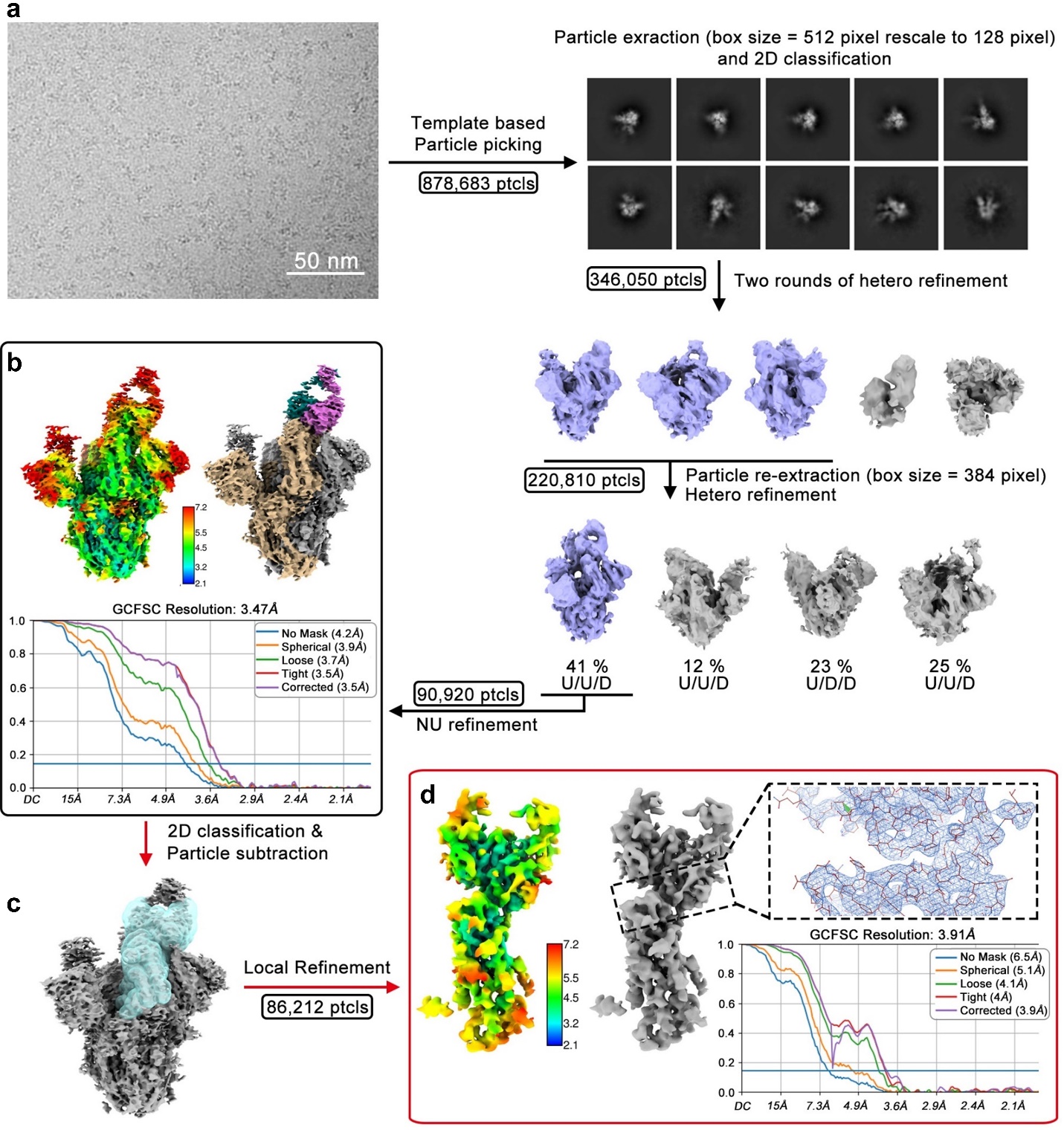


Figure. S5.

Flowchart depicting the cryo-EM data processing steps for the 7B3-spike whole map and 7B3/RBD locally refined map. **a** After 2D classification, classes with distinctive features were selected for subsequent 3D classification. Class colored purple was used for further classification, a class with the most particles and more completeness was selected for (**b**) further NU refinement and generated a map with an overall resolution of 3.5 Å and a local resolution about 5 Å in the region of the RBD and 7B3 interaction. **c** Local refinement strategy was adopted for further improving the resolution, one more round of 2D classification was used to clean particles and particle subtraction was performed to subtract the density in the core region of spike. A mask colored with transparent cyan that encompassing one RBD and mAb density was created to define the region for local refinement. **d** One round of local refinement was performed to generate the final local map. A zoomed-in view of 7B3 binding region and the FSC curves were shown aside.


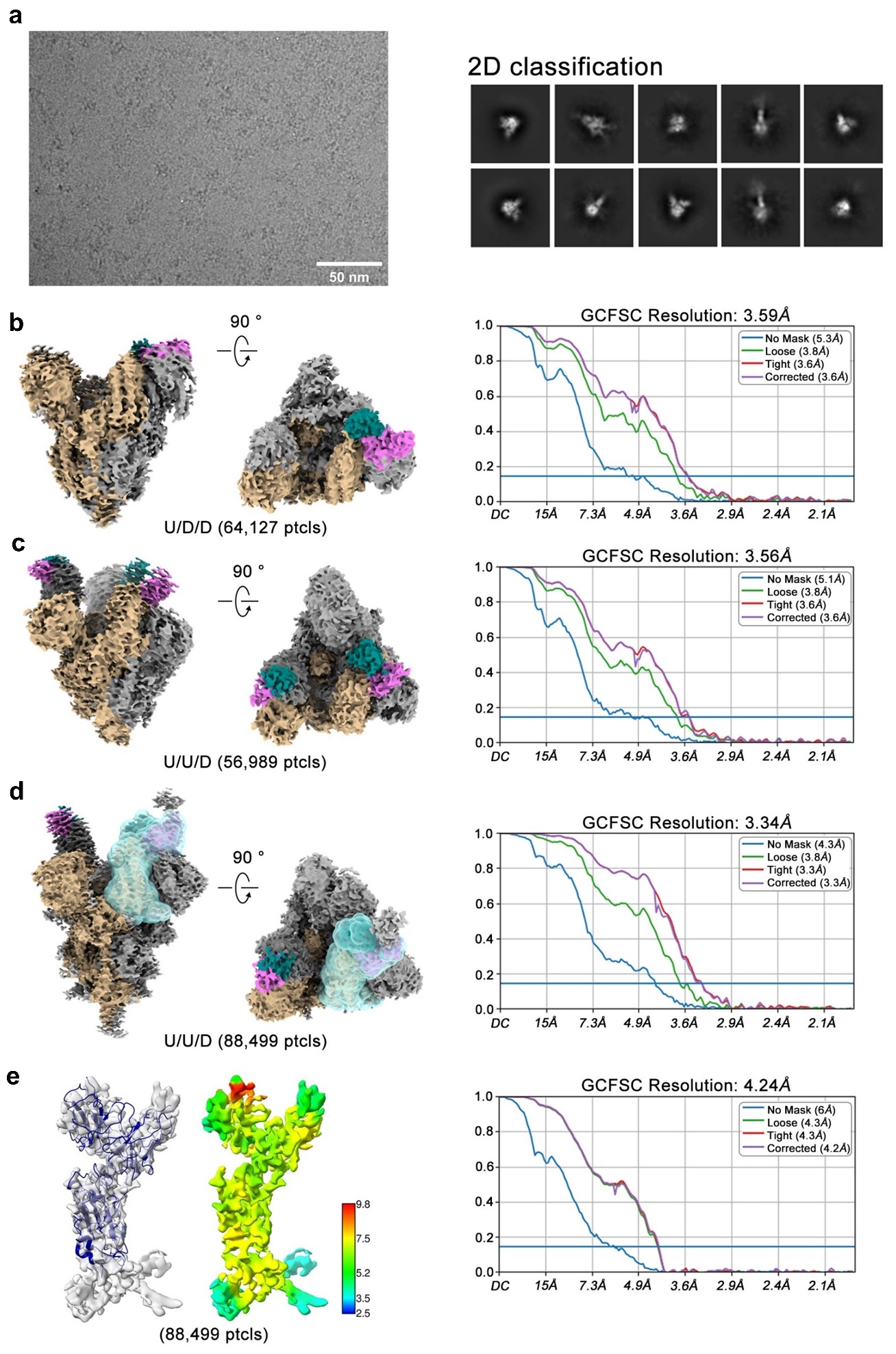


Figure. S6.

7B3 in complex with spike trimer of Omicron BA.1. **a** The incubated product was observed with (Left) Cryo-EM and (Right) the typical 2D classes were used to further 3D classification and refinement. Three refined maps were able to be recognized for the conformations of spike. Two orthogonal views of the map that adopt the U/D/D conformation (**b**), the U/U/D conformation (**c)** and the U/U/D conformation (**d**) with an overall resolution of 3.6 Å, 3.6 Å and 3.4 Å, respectively, and the corresponding FSC curves were listed aside. A transparent cyan mask in (**d**) indicating the region used for further local refinement. **e** Local refined map of the mask region in (**d**), The 7B3-RBD (WT) model was fitted well. However, due to the low resolution and large amount of missing detail in regions that RBD (WT) interact with 7B3, the map is insufficient for model building.


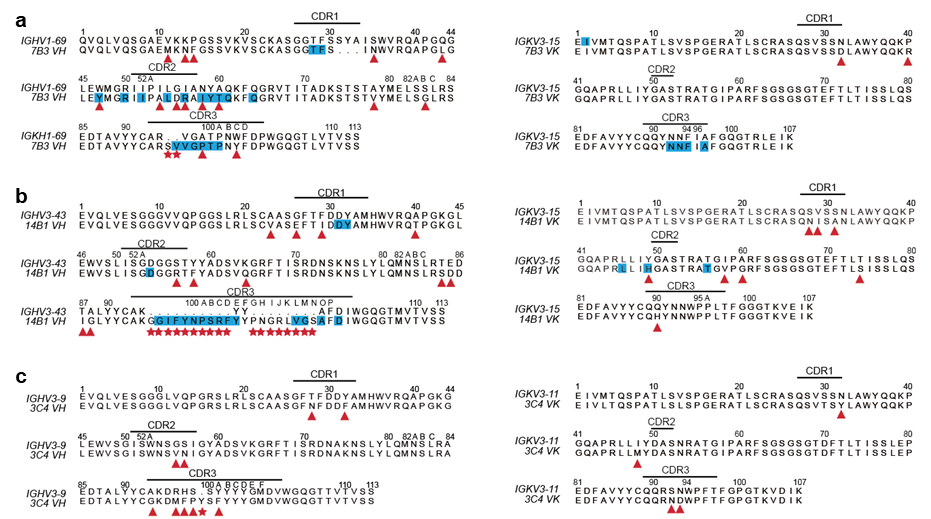


Figure. S7.

Amino acid sequence alignment of the variable heavy chain (VH) and light chain (VK) of 7B3 (**a**), 14B1 (**b**), and 3C4 (**c**) against the human germline. Nucleotide and amino acid sequences were analyzed by NCBI/IgBlast and IMGT/V-Quest to identify the germline V, D and J gene members with the highest sequence identity. Red triangles indicate positions of somatic hypermutation. Red stars mark the insertions at the junctions of rearrangement. The RBD-mAb interaction residues are boxed in blue. Absent residues are denoted with “.”. Consensus variable domain numbering is given above the aligned sequences by Kabat scheme. Complementarity determining regions (CDR1-3) are marked above the alignment.


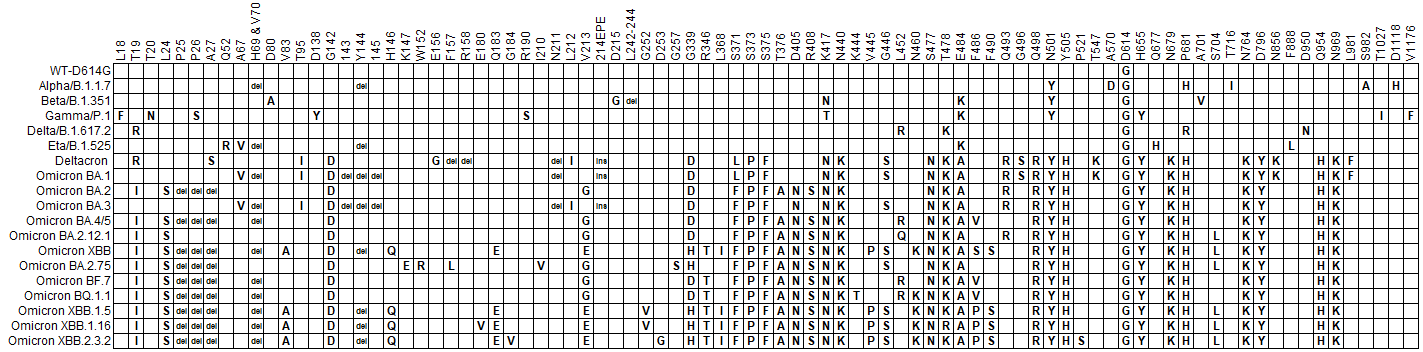


Figure. S8.

The amino acid alterations in the S protein of SARS-CoV-2 variants tested in this study.


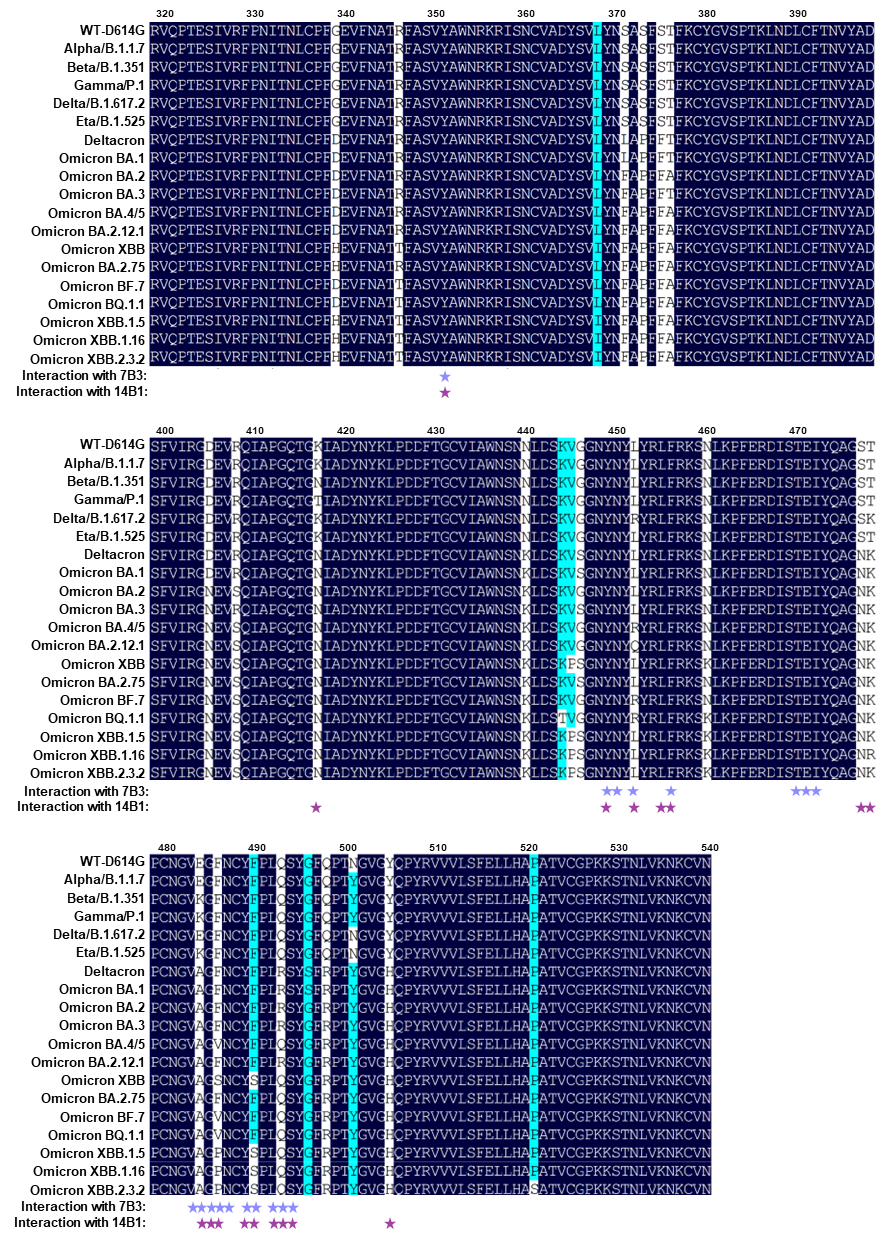


Figure. S9.

Amino acid sequence alignment of RBD from different SARS-CoV-2 variants. Blue stars mark the residues which contribute to the interaction between RBD and 7B3. Red stars mark the residues which contribute to the interaction between RBD and 14B1.

Table S1.

**Cryo-EM data collection, refinement, and model validation statistics**

|  | **RBD-14B1 complex PDB 8I3U EDB-35156** | | **RBD-7B3 complex PDB 8I3S EMD-35155** | | **Omicron BA.1 RBD-7B3 complex** |
| --- | --- | --- | --- | --- | --- |
| **Data collection and processing** | |  | |  | |
| Microscope | JEOL CRYO ARM 300 | JEOL CRYO ARM 300 | | JEOL CRYO ARM 300 | |
| Magnification | 50,000 × | 50,000 × | | 50,000 × | |
| Voltage (kV) | 300 | 300 | | 300 | |
| Frames per stack | 40 | 40 | | 40 | |
| Total dose per movie (e^-^/Å^2^) | 40 | 40 | | 40 | |
| Defocus range (μm) | -0.5 to -2.5 | -0.5 to -2.5 | | -0.5 to -2.5 | |
| Pixel size (Å) | 0.475 | 0.475 | | 0.475 | |
| Symmetry imposed | C1 | C1 | | C1 | |
| Final particle images (no.) | 488,934 | 86,212 | | 88,498 | |
| Map resolution (Å) | 3.6 | 3.9 | | 4.3 | |
| FSC threshold | 0.143 | 0.143 | | 0.143 | |
| **Model Refinement** |  |  | |  | |
| Model resolution (Å) | 3.6 | 3.9 | |  | |
| FSC threshold | 0.143 | 0.143 | |  | |
| Map sharpening B factor (Å^2^) | -128.3 | -135.6 | |  | |
| Model composition |  |  | |  | |
| Non-hydrogen atoms | 3,410 | 3,275 | |  | |
| Protein residues | 437 | 420 | |  | |
| Ligand | NAG:1 | NAG:1 | |  | |
| B factors(Å^2^)(mean) |  |  | |  | |
| Protein | 72.69 | 83.71 | |  | |
| Ligand | 98.31 | 98.37 | |  | |
| R.m.s. deviations |  |  | |  | |
| Bond length (Å) (# > 4σ ) | 0.002 (0) | 0.003 (0) | |  | |
| Bond angles (°) (# > 4σ ) | 0.528 (2) | 0.580 (2) | |  | |
| Validation |  |  | |  | |
| MolProbity score | 1.50 | 2.14 | |  | |
| Clash score | 8.69 | 14.59 | |  | |
| Poor rotamers (%) | 0.00 | 0.00 | |  | |
| Ramachandran plot |  |  | |  | |
| Favored (%) | 98.14 | 97.10 | |  | |
| Allowed | 1.86 | 2.90 | |  | |
| Disallowed | 0.00 | 0.00 | |  | |

Table S2.

**Summary of hydrogen bonding^*^ across the interface in 14B1-RBD and 7B3-RBD complexes**

| Hydrogen bond contacts between 14B1 and RBD | | | Hydrogen bond contacts between 7B3 and RBD | | |
| --- | --- | --- | --- | --- | --- |
| RBD | H | L | RBD | H | L |
| Tyr351(OH) | Arg100C(NH2) |  | Tyr449(O) | Thr28(OG1) |  |
| Lys417(NZ) | Asp31(OD2) |  | Glu484(OE1) | Tyr47(OH) |  |
| Ser477(OG) |  | Thr56(OG1) | Glu484(N) | Tyr59(O) |  |
| Glu484(OE1) | Tyr100E(OH) |  | Asn487(N) |  | Asn93(OD1) |
| Phe490(N,O) | Tyr99(OH) |  | Tyr489(OH) |  | Asn92(O) |
| Gln493(NE2) | Phe98(O) |  | Tyr489(OH) |  | Asn93(ND2) |
| Ser494(OG) | Asn100(O) |  | Phe490(N) | Pro99(O) |  |
| Ser494(OG) | Pro100A(O) |  | Ser494(N) | Val97(O) |  |
| Ser494(O) | Asn100(ND2) |  | Salt bridges | | |
| Tyr505(OH) | Asp53(OD2) |  | RBD | H | L |
|  |  |  | Glu484(OE2) | Arg50(NH1) |  |

^*^Hydrogen boding interactions were determined using LigPlot+ suite with a cutoff distance of 3.9 Å.

Table S3.

**Summary of van der Waals contacts^*^ across the interface in 14B1-RBD and 7B3-RBD complexes.**

| 14B1-RBD (van der Waals contacts) | | | 7B3-RBD (van der Waals contacts) | | |
| --- | --- | --- | --- | --- | --- |
| RBD | H | L | RBD | H | L |
| Lys417(2) | Asp31(2) |  | Tyr351(2) | Leu54(2) |  |
| Tyr449(2) | Pro100A(2) |  | Tyr449(5) | Thr28(1), Phe29(3), Val96(1) |  |
| Leu452(1) | Ser100B(1) |  | Asn450(4) | Thr28(4) |  |
| Leu455(3) | Asp31(2),Ile97(1) |  | Leu452(1) | Val97(1) |  |
| Phe456(2) | Ile97(2) |  | Phe456(1) | Pro99(1) |  |
| Ser477(1) |  | Thr56(1) | Thr470(7) | Arg56(7) |  |
| Thr478(2) |  | His49(2) | Glu471(2) | Arg56(2) |  |
| Glu484(3) | Tyr100E(2),Val100L(1) |  | Ile472(1) | Ile58(1) |  |
| Gly485(3) | Val100L(1),Gly100M(2) |  | Val483(12) | Tyr59(8), Thr60(2), Gln61(1), Gln64(1) |  |
| Phe486(19) | Asp101(5),Gly100M(2),Ala100O(4) | Leu46(2),His49(6) | Glu484(12) | Arg50(1), Ile58(2), Pro99(2)，Pro100A(2) | Phe94(5) |
| Tyr489(7) | Tyr32(1),Gly96(1),Ile97 (1),Tyr99(3),Val100L(1) |  | Gly485(13) |  | Phe94(13) |
| Phe490(12) | Tyr99(7),Phe100D(4), Tyr100E(1) |  | Phe486(19) |  | Ile2(6),Asn93(5),Phe94(7),Ala97(1) |
| Leu492(3) | Tyr99(2),Phe100D(1) |  | Asn487(3) |  | Asn93(3) |
| Gln493(5) | Ile97(3),Phe98(2) |  | Tyr489(18) | Pro99(5), Thr100(2) | Asn92(1),Asn93(10) |
| Ser494(10) | Asn100(5),Pro100A(2),Ser100B(3) |  | Phe490(17) | Arg50(3), Ile52(2), Ile58(2), Gly98(1), Pro99(9) |  |
| Tyr505(4) | Asp53(4) |  | Leu492(3) | Leu54(1), Gly98(2) |  |
|  |  |  | Gln493(3) | Val97(2), Pro99(1) |  |
|  |  |  | Ser494(4) | Val96(1),Val97(3) |  |

^*^Interactions were determined using LigPlot+ suite with a cutoff distance of 3.9 Å
